# Identifying and overcoming the sampling challenges in relative binding free energy calculations of a model protein:protein complex

**DOI:** 10.1101/2023.03.07.530278

**Published:** 2023-03-08

**Authors:** Ivy Zhang, Dominic A. Rufa, Iván Pulido, Michael M. Henry, Laura E. Rosen, Kevin Hauser, Sukrit Singh, John D. Chodera

**Affiliations:** 1 Computational and Systems Biology Program, Sloan Kettering Institute, Memorial Sloan Kettering Cancer Center, New York, NY 10065; 2 Tri-Institutional PhD Program in Computational Biology and Medicine, Weill Cornell Medical College, Cornell University, New York, NY 10065; 3 Tri-Institutional PhD Program in Chemical Biology, Weill Cornell Medical College, Cornell University, New York, NY 10065; 4 Vir Biotechnology, San Francisco, CA, USA

## Abstract

Relative alchemical binding free energy calculations are routinely used in drug discovery projects to optimize the affinity of small molecules for their drug targets. Alchemical methods can also be used to estimate the impact of amino acid mutations on protein:protein binding affinities, but these calculations can involve sampling challenges due to the complex networks of protein and water interactions frequently present in protein:protein interfaces. We investigate these challenges by extending a GPU-accelerated open-source relative free energy calculation package (Perses) to predict the impact of amino acid mutations on protein:protein binding. Using the well-characterized model system barnase:barstar, we describe analyses for identifying and characterizing sampling problems in protein:protein relative free energy calculations. We find that mutations with sampling problems often involve charge-changes, and inadequate sampling can be attributed to slow degrees of freedom that are mutation-specific. We also explore the accuracy and efficiency of current state-of-the-art approaches—alchemical replica exchange and alchemical replica exchange with solute tempering—for overcoming relevant sampling problems. By employing sufficiently long simulations, we achieve accurate predictions (RMSE 1.61, 95% CI: [1.12, 2.11] kcal/mol), with 86% of estimates within 1 kcal/mol of the experimentally-determined relative binding free energies and 100% of predictions correctly classifying the sign of the changes in binding free energies. Ultimately, we provide a model workflow for applying protein mutation free energy calculations to protein:protein complexes, and importantly, catalog the sampling challenges associated with these types of alchemical transformations. Our free open-source package (Perses) is based on OpenMM and available at https://github.com/choderalab/perses.

## INTRODUCTION

### Predicting the impact of amino acid mutations on protein:protein binding has important applications

Protein:protein interactions (PPIs) underlie fundamental biological processes, such as transcriptional regulation (e.g., p53:MDM2 [1]), signal transduction (e.g., GRB2-EGFR [2]), and membrane fusion (e.g., SARS-CoV-2 RBD:ACE2 [3]). As protein:protein interactions are driven by binding events, changes in protein:protein binding affinity often have functional impact. Even a single amino acid substitution can significantly alter binding and function, which can give rise to disease [4], impact the fitness of a pathogen [5], or alter the activity of monoclonal antibody drugs [6]. Thus, quantifying the impact of an amino acid mutation on protein:protein binding is highly useful for predicting the functional implication of a mutation, including providing mechanistic understanding of disease-associated genetic variants [7, 8] and facilitating the design of biologic drugs such as monoclonal antibodies [9, 10].

### Alchemical free energy calculations represent an accurate and generalizable approach for estimating mutational impact on PPIs

There are many experimental and computational approaches for quantifying the impact of a mutation on protein:protein binding. Experimental approaches, while highly accurate and generally considered “ground truth,” can be resource intensive, often requiring significant amounts of human labor time and costly reagents and instruments [11–14]. Computational methods, which can circumvent these resource challenges, serve as a complementary approach that can be combined with experimental methods to enable more efficient acquisition of high confidence data. Examples include computationally inexpensive methods (e.g., MM/PBSA and MM/GBSA [15, 16], machine learning (ML) models [17, 18], and Rosetta-based methods [19, 20]), as well as computationally expensive methods such as alchemical free energy calculations. Because they involve rigorous statistical mechanics and all-atom, explicit solvent simulations, alchemical free energy calculations are accurate and generalizable to any PPI with available structural data [21–24]. The optimal choice in approach will ultimately depend on the scientific goal and the computational resources available. However, given their accuracy and generalizability, as well as rapid advancements in graphics processing units (GPUs) that have made it feasible to carry out these calculations in reasonable timeframes [25–28], alchemical free energy calculations represent a highly promising approach for predicting mutational effect on protein:protein binding.

### Relative alchemical binding free energy calculations aim to predict the impact of a mutation on the free energy of binding $\left(\Delta \Delta G_{\text{binding}}\right)$

**Figure 1.**
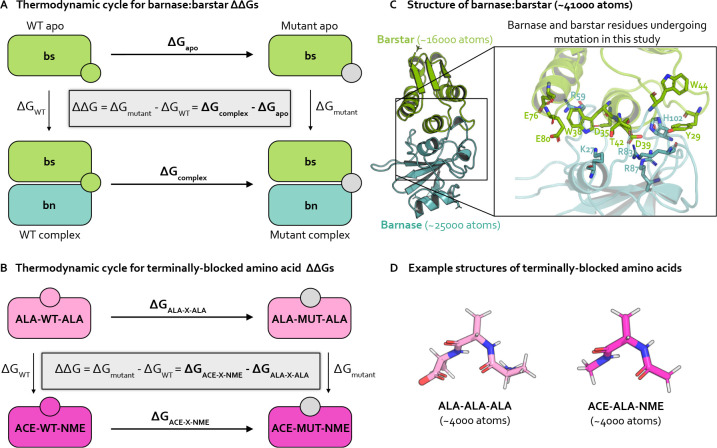
Relative free energy calculations predict the impact of single point mutations using thermodynamic cycles that each involve transformations in two environments. **(A)** Thermodynamic cycle representing how the relative binding free energy $\left(\Delta \Delta G_{\text{binding}}\right)$ can be computed for a protein mutation in the barnase:barstar complex. By cycle closure, the ΔΔG equation shown inside the thermodynamic cycle can be recovered. In practice, it is easier to compute the horizontal legs ($\Delta G_{\text{apo}}$ and $\Delta G_{\text{complex}}$, shown in bold) [32], which involve transforming a WT residue (green circle) into a mutant residue (gray circle). The free energy differences for each phase (apo and complex) are subtracted from each other to compute the $\Delta \Delta G_{\text{binding}}$. **(B)** Thermodynamic cycle representing how the relative free energy $(\left.\Delta \Delta G\right)$ can be computed for a protein mutation between two phases of terminally-blocked amino acids. The horizontal legs ($\Delta G_{ALA-X-ALA}$ and $\Delta G_{ACE-X-NME}$, shown in bold) are simulated, which involve transforming a WT residue (magenta or pink circle) into a mutant residue (gray circle). The free energy differences for each phase (ACE-X-NME and ALA-X-ALA) are subtracted from each other to compute the ΔΔG. **(C)** Structural model of barnase:barstar (PDB ID: 1BRS) with barstar shown in green and barnase shown in blue. Barstar and barnase contain ~16000 and ~25000 atoms, respectively (including hydrogens and solvent). Zoomed-in view of the barnase:barstar interface shows the 13 residues undergoing mutation in this study (all of which are interfacial) as sticks. Nitrogen atoms shown in blue and oxygen atoms are shown in red. **(D)** Example structural models of terminally-blocked amino acids: ALA-X-ALA and ACE-X-NME (where X is ALA) shown in pink and magenta, respectively. Each terminally-blocked amino acid contains ~4000 atoms (including hydrogens and solvent). Nitrogen atoms are depicted in blue, oxygen atoms in red, and hydrogen atoms in white.

**Figure 2.**
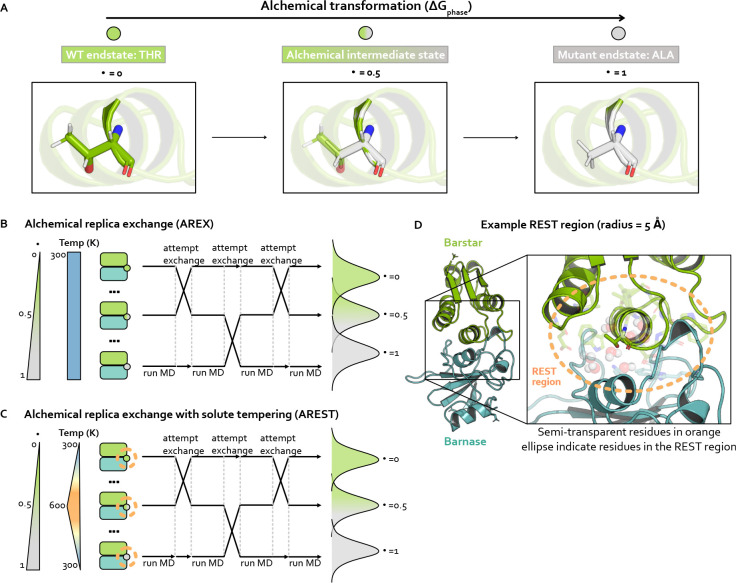
Strategies for sampling an alchemical transformation: Alchemical replica exchange (AREX) and alchemical replica exchange with solute tempering (AREST). AREST modifies AREX by introducing local heating around the alchemical region at intermediate alchemical states. **(A)** Schematic representing an alchemical transformation (with one alchemical intermediate state) for one simulation phase. The WT (λ=0, green) endstate contains a fully-interacting threonine residue and the Mutant (λ=1, gray) endstate contains a fully-interacting alanine residue. The alchemical intermediate (λ=0.5, green-gray gradient) state contains partially interacting threonine and alanine residues. Nitrogen atoms are shown in blue and oxygen atoms are shown in red. **(B)** Schematic representing alchemical replica exchange (AREX), sometimes called Hamiltonian replica exchange among alchemical states, which utilizes multiple replicas (in this schematic, three replicas) to explore alchemical states that bridge the WT (λ=0, green circle) and mutant (λ=1, gray circle) fully interacting states. The temperature remains constant at 300 K for all alchemical states. Representative configurational distributions for each alchemical state are shown (on the right) to be overlapping for neighboring states, which is a requirement for accurate ΔΔG estimates. **(C)** Schematic representing alchemical replica exchange with solute tempering (AREST), which elevates the effective temperature for a small region (i.e., the REST region) to further enhance sampling. The REST region is shown as an orange, dashed circle. The effective temperature of the REST region reaches a maximum at 600 K at λ=0.5. Representative configurational distributions for each alchemical state are shown (on the right) with less overlap than in AREX (panel B), because increasing the effective temperature usually causes increased thermodynamic length. **(D)** Structural model of barnase:barstar with barstar shown in green and barnase shown in blue. Zoomed-in view highlights an example REST region (orange, dashed circle) for residue T42 in barstar. T42 and neighboring residues (within 5 Å of T42) are shown as sticks and neighboring waters (also within 5 Å of T42) are shown as spheres.

While there are numerous types of alchemical free energy calculations [29], relative alchemical binding free energy (RBFE) calculations estimate the relative binding free energies $\left(\Delta \Delta G_{\text{binding}}\right.$) between two chemically similar complexes, e.g., protein:protein complexes that differ by an amino acid mutation (Figure 1 A). In protein mutation RBFE calculations [23, 24, 30, 31], the wild-type (WT) residue is transformed into the mutant residue through molecular dynamics simulations of alchemical (non-physical) intermediate states bridging the WT and mutant states (Figure 2A). This alchemical transformation is performed in two phases, complex and apo, which correspond to the mutating protein in the presence and absence of a protein binding partner, respectively (Figure 1A). The change in free energy associated with each phase $\left(\Delta G_{\text{phase}})\right.$ is estimated, and the difference in the $\Delta G_{\text{phase}}$ gives an estimate of the impact of the mutation on the binding free energy, $\Delta \Delta G_{\text{binding}}$ (Figure 1A).

### Achieving sufficient sampling of protein and water conformations is particularly challenging for RBFE calculations applied to protein:protein interactions

During the last couple of decades, RBFE calculations have become increasingly widely used in drug discovery projects for predicting the effects of small molecule modifications on protein:small molecule binding [33–39]. In comparison to small molecule transformations, application of RBFE calculations to protein mutations has been relatively limited, though recent studies have demonstrated that these methods can accurately predict mutational impact on protein:small molecule [30, 31, 40] and protein:protein binding [23, 24]. One reason for their lack of widespread use stems from the size of protein:protein complexes, which can frequently involve approximately double the number of atoms as are present in protein:small molecule complexes, making them more computationally expensive to simulate. However, the bigger hurdle has been the sampling challenges associated with alchemical transformations in protein:protein complexes [23, 41]. RBFE calculations require drawing decorrelated samples from the configurational probability distributions at each alchemical state [42], a nontrivial task for protein:protein complexes because the energy landscapes often contain many minima which can give rise to slow degrees of freedom. Slow degrees of freedom are more prevalent in protein:protein complexes because protein:protein interfaces are generally broader than protein:small molecule interfaces and typically involve complex protein and water interaction networks [4, 43]. Upon mutation, extensive reorganization of the mutating residue along with its closely-packed neighborhood of interfacial protein residues and waters may be required before one can draw decorrelated samples.

### Pinpointing slow degrees of freedom can help address the sampling problems in protein:protein RBFE calculations, but existing approaches are not automated

Determining the slow degrees of freedom causing sampling problems in alchemical free energy calculations may enable improved sampling via application of methods that accelerate known slow degrees of freedom (e.g., metadynamics [44, 45], umbrella sampling [46], adaptive biasing force [47]). Moreover, identifying slow degrees of freedom helps enumerate the common challenges associated with alchemical transformations and the limitations of existing methods, which will facilitate the improvement of existing methods or the design of new ones. However, pinpointing the slow degrees of freedom in protein:protein interfaces can be challenging because it typically involves careful manual inspection of simulation trajectories [32, 48, 49], a process which requires biophysical intuition and can be tedious even for experienced practitioners. Moreover, the manual inspection approach is not scalable as examining the trajectories for tens or hundreds of mutations would be extremely impractical.

Here, we investigate sampling problems associated with protein mutation relative free energy calculations using (1) terminally-blocked amino acids, a small and simple test system relatively free of interfacial complexities and (2) barnase:barstar, a well-studied protein:protein complex. We augment an existing open-source relative free energy calculation package (Perses [50], https://github.com/choderalab/perses) to carry out these calculations and describe experiments and automated analyses that identify likely causes of sampling problems in an automated fashion. We find that sampling challenges are more likely to occur for charge-changing mutations and can be attributed to mutation-dependent slow degrees of freedom. We also compare the accuracy and efficiency of state-of-the-art enhanced sampling approaches—alchemical replica exchange (AREX) [28, 51, 52] and alchemical replica exchange with solute tempering (AREST) [53, 54]—for overcoming the sampling challenges. We find that given sufficient simulation time, our predictions are accurate with respect to experiment (RMSE 1.61, 95% CI: [1.12, 2.11] kcal/mol), with 86% of predictions lying within 1 kcal/mol of experimental $\Delta \Delta G_{\text{binding}}s$ and 100% of predictions having the correct sign.

## THEORY

We perform alchemical free energy calculations using two state-of-the-art enhanced sampling approaches: (1) alchemical replica exchange (AREX) [28, 51, 52], the current recommended approach based on best practices [22] and (2) alchemical replica exchange with solute tempering (AREST) [53, 54], a sampling scheme which builds upon AREX by increasing the temperature of a region around the mutating residue and has been shown to improve sampling over AREX for some transformations [54–56]. Here, we give a brief overview of the salient aspects of each method, as well as the general approach we take to alchemical free energy calculations for protein mutations. The alchemical approach is implemented in an open-source package (Perses [50], available at https://github.com/choderalab/perses). Complete simulation details can be found in the **Detailed Methods**.

### Alchemical transformation

Alchemical free energy calculations aim to sample a set of alchemical states which are defined such that two endstates of interest are bridged by alchemical intermediate states with modified Hamiltonians. The Hamiltonians are modified such that the nonbonded interactions (and potentially valence terms) of the WT and mutant residues are gradually transformed between interacting and non-interacting. The first alchemical state, called the WT endstate, typically involves the WT residue fully interacting with its environment and the mutant residue completely non-interacting. The last alchemical state, known as the mutant endstate, involves the mutant residue fully interacting and the WT residue non-interacting. A series of intermediate alchemical states bridging the endstates is defined such that WT and mutant residues are partially interacting with their environments to varying extents. Taken together, these alchemical states form the alchemical transformation (Figure 2A). Note that amino acids with backbone cycles (such as proline) require a modified version of this approach to avoid the noninteracting residue influencing the conformational distribution of fully interacting residues [57], but the mutations in this study do not involve this type of amino acid.

To characterize an alchemical transformation, we need to specify both *which* interactions will be alchemically modified during the transformation and *how* we will modify them. We first identify the alchemical interactions by defining an atom mapping, which exploits the partial similarity in WT and mutant topologies by pairing up the WT and mutant atoms that will share coordinates. The atom mapping is then used to classify each atom into an “atom class”: “unique old” atoms are unmapped atoms that are only present in the WT residue, “unique new” atoms are unmapped atoms that are only present in the mutant residue, “core” atoms are mapped atoms that are shared between the WT and mutant residues, and “environment” atoms are mapped atoms that are shared between the topologies but lie outside of the WT and mutant residues. Interactions involving “unique old”, “unique new” and “core” atoms are considered alchemical interactions. Since increasing the number of alchemical interactions also increases the thermodynamic length (i.e., the distance between alchemical states) [58], an atom mapping should be defined to maximize the number of atoms mapped between the two residues, which minimizes thermodynamic length. An optimal mapping finds a balance between minimizing thermodynamic length and taking advantage of the built-in enhanced sidechain sampling that occurs when the unmapped atoms are non-interacting (i.e., the nonbonded interactions are scaled to zero, so the sidechains can more easily sample alternate rotameric states). To this end, we chose an atom mapping that maps all atoms between the WT and mutant residues up to and including the beta carbon (but not including beta hydrogens). Because we constrain bonds to hydrogen, we un-map any hydrogen atoms whose bond lengths would change between WT and mutant.

To specify how the energies should be modified during the alchemical transformation, we introduce an alchemical parameter λ∈[0,1] into the potential energy function U(x), forming the alchemical potential energy function U(x;λ). The alchemical potential U(x;λ) is typically evaluated at a different λ value for each alchemical state. For the WT endstate, U(x;λ=0) is identical to the unmodified WT potential with the addition of the standard valence terms (and not the nonbonded interactions) of the unique new atoms (sometimes called “dummy” atoms). For the mutant endstate, U(x;λ=1) is identical to the unmodified mutant potential, but with unique old atoms as noninteracting dummy atoms that only retain their valence terms. For the alchemical intermediate states (λ∈(0,1)), U(x;λ) is a modified potential where interactions involving the WT and mutant residues are scaled to varying extents. The set of λ values sampled in the alchemical transformation is termed the “alchemical protocol”. To define the alchemical protocol for this study, we selected evenly spaced λ values from a simple linear function (Supplementary Figure 1A). The number of λ values used for each calculation was chosen such that the neighboring alchemical states have good phase space overlap (for more details, see **Detailed Methods**), which permits robust estimation of free energy differences between the fully-interacting WT and mutant endstates [59, 60].

Using the alchemical parameter λ, we define the potential energy functions for electrostatic and steric interactions. We compute the alchemically modified electrostatics interaction energy according to the Particle Mesh Ewald (PME) method [61] with linearly interpolated charges. For the direct space electrostatics contribution:

(1) $$U_{\text{direct}\;}(r;\lambda )\;=C\frac{q_{i}(\lambda )q_{j}(\lambda )}{r_{\text{eff}\;}(r,\lambda )}\cdot erfc\left(\alpha r_{\text{eff}\;}(r,\lambda )\right)\;\;q_{i}(\lambda )\;=\chi_{i}^{\text{old}\;}q_{i}^{\text{old}\;}(\lambda )+\chi_{i}^{\text{new}\;}q_{i}^{\text{new}\;}(\lambda )+\chi_{i}^{\text{core}\;}\left(q_{i}^{\text{old}\;}(\lambda )+q_{i}^{\text{new}\;}(\lambda )\right)+\chi_{i}^{\text{env}\;}q_{i}^{\text{old}\;}\;\;q_{i}^{\text{old}\;}(\lambda )\;=(1-\lambda )q_{i}^{\text{old}\;}\;\;q_{i}^{\text{new}\;}(\lambda )\;=\lambda q_{i}^{\text{new}\;}$$

where α is an internal PME parameter (calculated based on the PME error tolerance and the cutoff distance, with dimension 1/length), C is the Coulomb constant (with dimension energy/length^2^), $q_{i}(\lambda )$ and $q_{j}(\lambda )$ are the functions for computing the potentially alchemically modified charges of atoms i and j (with dimension of charge), and $q_{i}^{\text{old}}$ and $q_{i}^{\text{new}}$ are the charges of atom i in the old topology and new topology, respectively $\chi_{i}^{\text{old}},\;\chi_{i}^{\text{new}},\;\chi_{i}^{\text{core}}$, and $\chi_{i}^{\text{env}}$ are indicator functions denoting whether atom i belongs in the unique old, unique new, core, and environment atom classes, respectively.

$r_{\text{eff}}(r,\lambda )$ denotes the *effective interaction distance* used for computing the interaction energy, and depends on both the actual inter-particle separation r and the alchemical parameter λ. To avoid singularities in the computation of electrostatics (and sterics) energies, we use a softcore approach that involves “lifting” certain inter-atomic distances into the “4th dimension”, inspired by work of Pomès [62]:

(2) $$r_{eff}(r,\lambda )\;=\sqrt{r^{2}+w(\lambda {)}^{2}}\;\;w(\lambda )\;=w_{\text{lifting}\;}\cdot \left(\chi_{ij}^{\text{old}\;}\lambda +\chi_{ij}^{\text{new}\;}(1-\lambda )\right)\;\;\chi_{ij}^{old\;}\;=\left\{\begin{array}{ll} 1 & \chi_{i}^{\text{old}\;}+\chi_{j}^{\text{old}\;}\geq 0\\ 0 & \;else\; \end{array}\right.\;\;\chi_{ij}^{\text{new}\;}\;=\left\{\begin{array}{ll} 1 & \chi_{i}^{\text{new}\;}+\chi_{j}^{\text{new}\;}\geq 0\\ 0 & \;else\; \end{array}\right.$$

where r is the distance between atoms i and j,w(λ) is the function for computing the lifting distance, and $w_{\text{lifting}}$ is the maximal lifting distance, which was selected to minimize the number of alchemical states needed to produce robust free energy estimates by maintaining good overlap among neighboring alchemical states. $\chi_{ij}^{\text{old}}$ is an indicator function that assumes the value of unity only when at least one of the atoms (i and j) belongs in the unique old atom class (and zero otherwise), and $\chi_{ij}^{\text{new}}$ is an indicator function indicating whether at least one of the atoms (i and j) belongs in the unique new atom class. For more details on this approach, see **Detailed Methods**.

We compute the PME reciprocal space and self-energy contributions using the default energy functions in OpenMM [26], but with linearly interpolated charges, where interpolation was performed in the same manner as was done for the direct space.

We compute the alchemically modified sterics interaction energy as a standard Lennard-Jones 12–6 potential [63–65] with linearly interpolated *σ* and *ϵ* and “lifted” interaction distances to create a softcore potential:

(3) $$U_{\text{sterics}\;}(r;\lambda )\;=4\;\epsilon_{ij}(\lambda )x(x-1.0);x={\left(\frac{\sigma_{ij}(\lambda )}{r_{\text{eff}\;}(r,\lambda )}\right)}^{6}\;\;\sigma_{ij}(\lambda )\;=\frac{\sigma_{i}(\lambda )+\sigma_{j}(\lambda )}{2};\sigma_{i}(\lambda )=\chi_{i}^{\text{old}\;}\sigma_{i}^{\text{old}\;}+\chi_{i}^{\text{new}\;}\sigma_{i}^{\text{new}\;}+\chi_{i}^{\text{core}\;}\left((1-\lambda )\sigma_{i}^{\text{old}\;}+\lambda \sigma_{i}^{\text{new}\;}\right)+\chi_{i}^{\text{env}\;}\sigma_{i}^{\text{old}\;}\;\;\epsilon_{ij}(\lambda )\;=\sqrt{\epsilon_{i}(\lambda )\cdot \epsilon_{j}(\lambda )};\epsilon_{i}(\lambda )=\chi_{i}^{\text{old}\;}\epsilon_{i}^{\text{old}\;}(\lambda )+\chi_{i}^{\text{new}\;}\epsilon_{i}^{\text{new}\;}(\lambda )+\chi_{i}^{\text{core}\;}\left(\epsilon_{i}^{\text{old}\;}(\lambda )+\epsilon_{i}^{\text{new}\;}(\lambda )\right)+\chi_{i}^{\text{env}\;}\epsilon_{i}^{\text{old}\;}(\lambda )\;\;\epsilon_{i}^{\text{old}\;}(\lambda )\;=(1-\lambda )\epsilon_{i}^{\text{old}\;};\epsilon_{i}^{\text{new}\;}(\lambda )=\lambda \epsilon_{i}^{\text{new}\;}$$

Here, $\sigma_{ij}(\lambda )$ is the function for computing the potentially alchemically modified distance at which the interaction energy crosses zero for atoms i and $j.\;\sigma_{i}^{\text{old}\;}$ and $\sigma_{i}^{\text{new}\;}$ are the distances at which the energy equals zero for atom i in the old topology and new topology, respectively. $\epsilon_{ij}(\lambda )$ is the function for computing the potentially alchemically modified interaction strength for atoms i and $j.\;\epsilon_{i}^{\text{old}\;}$ and $\epsilon_{i}^{\text{new}\;}$ are the interaction strengths for atom i in the old topology and new topology, respectively.

**Figure 3.**
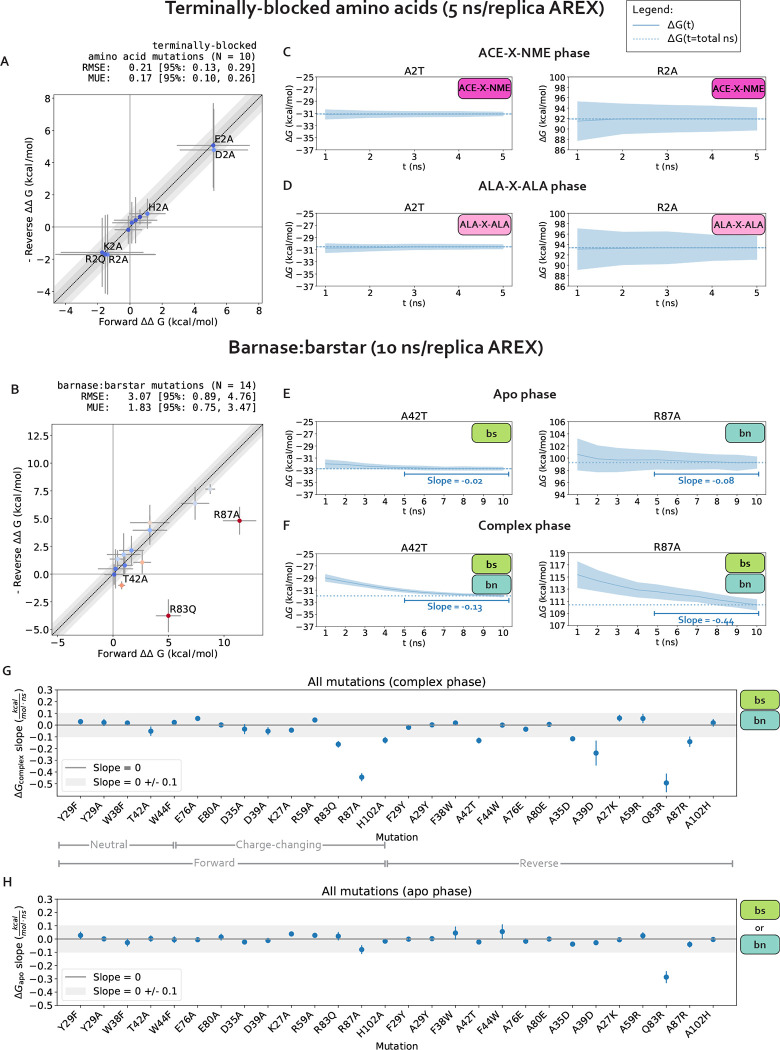
The relative free energy (ΔΔG) predictions for small terminally-blocked amino acid mutations are internally consistent and show good convergence, but several of the predictions for interfacial barnase:barstar mutations show poor internal consistency due to slow convergence of complex phase free energy differences $\left(\Delta G_{complex}s\right)$. **(A)** Forward versus (negative of the) reverse ΔΔGs for each terminally-blocked amino acid mutation computed using alchemical replica exchange (AREX) simulations (number of states = 12 and 24 for neutral and charge mutations, respectively, and simulation time = 5 ns/replica for each phase). Data points are labeled if the mutation involves a charge-change (to emphasize that our counterion introduction scheme works well in the absence of sampling problems). **(A)** (continued) The y = x (black dotted) line represents zero discrepancy between forward and (negative of the) reverse ΔΔG, the dark gray shaded region represents 0.5 kcal/mol discrepancy, and the light gray region represents 1 kcal/mol discrepancy. Data points are colored by how far they are from zero discrepancy (dark blue and red indicate close to and far from zero, respectively). Error bars represent two standard errors and were computed by bootstrapping the decorrelated reduced potential matrices 200 times. Root mean square error (RMSE) and mean unsigned error (MUE) are shown with 95% confidence intervals obtained from bootstrapping the ΔΔGs 1000 times. **(B)** Forward versus (negative of the) reverse ΔΔGs for each barnase:barstar mutation computed using alchemical replica exchange (AREX) simulations (number of states = 24 and 36 for neutral and charge mutations, respectively and simulation time = 10 ns/replica for each phase). Data points are labeled if the 95% CIs of the forward and (negative of the) reverse $\Delta \Delta G_{\text{binding}}s$ are not within 1 kcal/mol of each other. For more details on the plot and error bars, refer to the caption for panel A. **(C)** Free energy difference (ΔG) time series for representative mutations A2T (left) and R2A (right) in the ACE-X-NME phase. Alchemical replica exchange simulations were performed with number of states = 12 and 24 for A2T and R2A, respectively and the simulation time was 5 ns/replica. Dashed line indicates the ΔG at t = 5 ns. Shaded region represents ± two standard errors, which were computed by bootstrapping the decorrelated reduced potential matrices 200 times. **(D)** Same as (B), but for the ALA-X-ALA phase, instead of the ACE-X-NME phase. **(E)** Free energy difference (ΔG) time series for the apo phase of representative mutations with sampling problems: A42T (left) and R87A (right). Alchemical replica exchange simulations were performed with number of states = 24 and 36 for A42T and R87A, respectively and the simulation time was 10 ns/replica. Dashed line indicates the ΔG at t = 10 ns. For details on the error bars, refer to the caption for panel C. **(F)** Same as (E), but for the complex phase instead of the apo phase. **(G)** Slopes of the last 5 ns of the $\Delta G_{complex}$ time series for each barnase:barstar mutation are shown as blue circles. $\Delta G_{complex}$ time series were generated from complex phase AREX simulations (number of states = 24 and 36 for neutral and charge mutations, respectively, and simulation time = 10 ns/replica). Error bars represent 2 standard errors and were computed using the SciPy linregress function. Slopes within error of the shaded gray region (0 ± 0.1 kcal/mol/ns) are close to zero and are therefore considered “flat.” **(H)** Same as (G), but for apo phase barnase or barstar mutations instead of complex phase barnase:barstar mutations.

For charge-changing mutations, we ensure the system remains electrostatically neutral by transforming a water molecule in the WT system into a sodium or chloride ion in the mutant system. The ΔΔGs for charge-changing mutations in terminally-blocked amino acids are internally consistent, indicating that in the absence of sampling problems, our counterion scheme enables robust estimation of free energies (Figure 3A). Further details on this implementation can be found in **Detailed Methods**.

We estimate free energy differences using the Multistate Bennett Acceptance Ratio (MBAR), which is an asymptotically unbiased estimator that, in the large sample limit, often has lower variance compared to other commonly used estimators [60].

### Alchemical replica exchange (AREX)

Alchemical free energy calculations must sample from a chain of alchemical intermediate states bridging the two endstates of interest (Figure 2A). Because the introduction or deletion of bulky residues can often frustrate sampling within alchemical states in which these residues are almost fully interacting, alchemical free energy calculations often use replica exchange simulations to help reduce correlation times and overcome sampling challenges. Replica exchange enhances sampling by allowing each replica to visit multiple alchemical states, including those states which may help more rapidly decorrelate slow degrees of freedom because of their modified Hamiltonians [28, 51, 52]. Here, we refer to this approach as alchemical replica exchange (AREX).

AREX can be thought of as a Markov Chain Monte Carlo (MCMC) algorithm that aims to generate equilibrium samples from a family of K probability densities corresponding to the K alchemical states:

(4) $$x_{k}\sim p\left(x_{k}|s_{k}\right)\propto exp\left[-u_{s_{k}}(x)\right]k=1,\ldots ,K$$

where $x_{k}$ is a configuration drawn from state $k,\;s_{k}$ is the $k^{\text{th}\;}$ state, and $u_{s_{k}}(x)$ is the potential energy of sample x at state $s_{k}$.

To generate equilibrium samples, AREX utilizes weakly-coupled replicas (copies of the system of interest), where the number of replicas is typically equal to the number of alchemical states K. AREX employs a Gibbs sampling framework where in each iteration n, the positions $X_{n}={\left\{x_{k}\right\}}_{k=1}^{K}$ of all K replicas are first updated with molecular dynamics simulations, yielding $X_{n+1}$, and then the permutation set of alchemical state indices $S_{n}={\left\{s_{k}\right\}}_{k=1}^{K}$ associated with the corresponding positions are updated based on the updated positions $X_{n+1}$, yielding $S_{n+1}$:

(5) $$X_{n+1}\sim P\left(X_{n+1}|X_{n},S_{n}\right)\;S_{n+1}\sim P\left(S_{n+1}|X_{n+1}\right)$$

In sufficiently long simulations, the resulting samples $\left(X_{n},S_{n}\right)$ are distributed with respect to the joint probability density $P\left(X_{n},S_{n}\right)$ such that

(6) $$P(X,S)\equiv \prod_{k=1}^{K}\;p\left(x_{k}|s_{k}\right)$$

The algorithm updates the state indices by exchanging the alchemical state labels for pairs of replicas according to a Metropolis criterion that compares the energies of the two replicas considered for swapping. The replica swap acceptance rate will depend on how well the alchemical states overlap, i.e., the thermodynamic length between states (Figure 2B). Numerous methods can be used to update the states S, including attempting exchanges only between replicas visiting neighboring thermodynamic states (where state overlap is highest). Here, we used a simple strategy that attempts to draw an independent permutation $S_{n+1}$ given configuration $X_{n+1}$ by attempting many swaps of pairs of alchemical state indices, which has been shown to enhance mixing and reduce correlation times [66].

AREX involves running many cycles of molecular dynamics followed by exchange attempts with the goal of ensuring all replicas ultimately perform a random walk through all alchemical states (Figure 2B). If the exchanges are accepted at a sufficiently high rate over the course of the simulation, we expect to observe improved sampling because configurational correlation times associated with the alchemical region are likely decreased for alchemical states with partially interacting residues.

### Alchemical replica exchange with solute tempering (AREST)

AREST is AREX with an added layer of sophistication that aims to enhance sampling to a greater extent than AREX. AREST involves running AREX with a REST (replica exchange solute tempering [53]) region, a user-defined set of atoms for which the effective temperature is increased in alchemical intermediate states (Figure 2C–D). Therefore, in AREST, the alchemical states do not solely differ by the extent to which the WT and mutant residues are interacting with their environment, they also differ by the effective temperature of the REST region. Although introducing differences in effective temperature will increase the thermodynamic length between alchemical states (Figure 2C), the goal is to decrease the correlation time of the slowest degrees of freedom sufficiently to compensate for the decrease in state overlap, yielding more decorrelated samples in the same amount of total simulation time.

To incorporate REST into AREX, we classify each bond, angle, torsion, and nonbonded interaction as “REST” (all atoms in the interaction are part of the REST region), “inter” (at least one atom is part of the REST region and at least one atom is not), or “non-REST” (none of the atoms are part of the REST region) based on an initial conformation. Each interaction energy is multiplied by a scale factor depending on the REST class. Therefore, total potential energy is defined as:

(7) $$u_{\text{total}\;}(\lambda )\;=\alpha (\lambda )u_{\text{rest}\;}(\lambda )+\sqrt{\alpha (\lambda )}u_{\text{inter}\;}(\lambda )+u_{\text{nonrest}\;}(\lambda )\;\;\alpha (\lambda )\;\sim \lambda \left(T_{0}/T_{max}\right)$$

where $T_{0}$ is the temperature of the desired distribution and $T_{max}$ is the user-selected maximum effective temperature. We set the REST scaling factor α(λ) to be dependent on the alchemical coordinate λ where α(0)=α(1)=1 to ensure the endstates are unscaled. The function we used to define the REST scale factor α(λ) is given in Supplementary Figure 1B.

## METHODS AND SYSTEMS

For complete details on system setup and simulation parameters, see **Detailed Methods**.

### Barnase:barstar

Our investigation primarily focuses on the bacterial protein:protein complex barnase:barstar. The interaction of barnase, an extracellular ribonuclease, with its intracellular inhibitor, barstar, regulates RNA degradation in bacterial cells with a binding free energy of −19 kcal/mol [67]. Solvated barnase:barstar simulation models contain only ~41,000 atoms (including hydrogens and solvent), making it a computationally tractable system for studying sampling challenges in high-affinity protein:protein interfaces (Figure 1C, see **Detailed Methods** for system preparation details). Barnase:barstar has been well-studied both computationally [24, 41, 68] and experimentally [67, 69–71]). The barnase:barstar mutations considered in this work come from Schreiber et al. [67], who used stopped-flow measurements to derive experimental relative binding free energies $\left(\Delta \Delta G_{\text{binding}}\right)$ for 14 single amino acid substitutions across 13 residue positions in either barnase or barstar. The $\Delta \Delta G_{\text{binding}}s$ for this set of mutations span an unusually large dynamic range (7.8 kcal/mol) with a statistical error of 0.1 kcal/mol, and involves a diverse set of amino acids, making it particularly useful for assessing quantitative predictive models. All mutations occur within or are in close proximity to the barnase:barstar interface, which is a complex network of interactions dominated by electrostatic interactions and coordinated by buried waters (Figure 1C) [67]. Therefore, the mutations tends to disrupt numerous interfacial interactions, potentially requiring significant conformational and water reorganization to achieve equilibrium, which may give rise to sampling challenges.

### Terminally-blocked amino acids

As a control to the complexity of barnase:barstar, we also study terminally-blocked amino acids, which lack the complex interaction networks of barnase:barstar and have relatively few solute degrees of freedom. Specifically, we introduce mutations in small, solvated amino acids in two different environments: either terminally-blocked with ACE and NME caps at the N- and C-termini, respectively (ACE-X-NME), or terminally blocked with ALA residues with natural zwitterionic termini (ALA-X-ALA) (Figure 1D). The terminally-blocked mutation set consists of the same amino acid mutations as in the barnase:barstar mutation set, but contains only 10 total mutations (instead of 14 for barnase:barstar) as some of the barnase:barstar mutations involve the same amino acid transformation at different residue positions. By introducing the same mutations into the terminally-blocked amino acids, we separate the sampling challenges present in the barnase:barstar interface from the common challenges associated with alchemical free energy calculations.

To obtain relative free energies (ΔΔGs), we estimate the free energy differences (ΔGs) for two phases. For barnase:barstar, we are interested in the $\Delta \Delta G_{\text{binding}}$, so the two simulation phases are complex and apo (Figure 1A). For terminally-blocked amino acids, there is no notion of binding, so the two phases are: ACE-X-NME and ALA-X-ALA (Figure 1B).

## RESULTS

In the following sections, we investigate the sampling problems associated with applying relative free energy calculations to predict the impact of mutations in a model protein:protein complex. We establish an open-source workflow which consists of: (1) identifying mutations that are potentially plagued by sampling problems, (2) determining the slow degrees of freedom responsible for poor sampling, and (3) exploring state-of-the-art approaches for improving sampling. We first apply our workflow to a simple test system, terminally-blocked amino acids. We then focus the rest of the work on sampling challenges at the complex protein:protein interface of barnase:barstar, benchmarking to experimentally-determined binding free energy.

### 1 Mutations at protein:protein interfaces can be challenging for alchemical replica exchange relative free energy calculations, likely due to inadequate sampling in complex phase simulations

#### 1.1 The relative free energies (ΔΔGs) for terminally-blocked amino acid mutations are internally consistent, well converged, and relatively absent of sampling problems

We first establish that running alchemical replica exchange (AREX) with our alchemical approach (e.g., alchemical protocol, atom mapping, softcore and counterion approaches, etc.) is free of sampling and convergence issues when the mutation is not located in the context of a complex network of protein interactions. We estimated the ΔΔGs of 10 terminally blocked amino acid mutations–between two environments: ACEX-NME and ALA-X-ALA, where X is an amino acid–in both the forward (A→B) and reverse (B→A) directions. A mutation was considered internally consistent if the ΔΔG for the forward mutation (A→B) was within statistical error of the -ΔΔG for the reverse mutation (B→A). We found that with 5 ns/replica AREX simulations, all of the mutations are internally consistent and the forward ΔΔGs match the negative of the reverse ΔΔGs with high accuracy (root mean square error (RMSE): 0.21, 95% confidence interval (Cl): [0.13, 0.29] kcal/mol, Figure 3A).

We next confirm that our calculations lack replica mixing bottlenecks and convergence issues. We first checked that there are no replica mixing bottlenecks for any of the mutations, indicating that the alchemical states are spaced such that they have reasonable overlap (Supplementary Figure 2). We next determined the extent to which the free energy difference of mutating WT→ Mutant in one phase (ΔG) is converged because a converged ΔG indicates that the simulation has likely sampled all relevant degrees of freedom sufficiently. We assessed convergence by monitoring the changes in ΔG as a function of simulation time, which we call a “ΔG time series”. A ΔG time series was considered converged if, within the last five nanoseconds, it appeared flat with a close-to-zero slope (0 ± 0.1 kcal/mol/ns) (Supplementary Figure 3A). A ΔG time series was considered not converged if the magnitude of the slope of the last 5 ns was not within statistical uncertainty of 0 kcal/mol/ns. We found that for all 10 mutations in both phases and in both the forward and reverse directions, the slope of the ΔG time series is within statistical uncertainty of 0 kcal/mol/ns (Figure 3C–D, Supplementary Figure 3B–C), suggesting that the calculations are converged and relatively free of sampling problems.

Finally, we verify that 5 ns/replica AREX simulations thoroughly sample the slowest degrees of freedom for terminally blocked amino acid mutations [72]. We monitored the *ϕ* and *Ψ* angles for the ACE-X-NME phase of two representative mutations with significant sampling problems in barnase:barstar, A2T (ALA to THR at residue 2) and R2A (ARG to ALA at residue 2) (see Section 2). If the *ϕ* and *Ψ* degrees of freedom are thoroughly sampled, the time series should rapidly decorrelate. We quantify the extent to which each time series is hindered by slow correlation times by estimating its statistical inefficiency, g = 2*τ* +1, which is proportional to the autocorrelation of the time series *τ* [73, 74]. If the statistical inefficiency (g) is close to 1, the samples are completely decorrelated at the sampling interval (here, 1 ps), and the larger the value of g, the more correlated the samples are [74]. We observed that for both representative mutations, ACE-X-NME phase simulations thoroughly sample both angles with g close to 1 (Supplementary Figure 4), providing further support that the terminally-blocked amino acid calculations are converged and have minimal sampling problems.

These results demonstrate that in the absence of significant sampling problems, running AREX with our alchemical approach can provide reliable estimates of relative free energies (ΔΔGs), indicating that we can use this approach to explore the challenges associated with applying RBFE calculations to interfacial residues in the barnase:barstar protein:protein complex.

#### 1.2 Several barnase:barstar mutation predictions show poor accuracy due to slow convergence of the complex phase free energy difference $\left(\Delta G_{complex}\right)$, suggesting the presence of sampling problems

We assess the performance of AREX on predicting barnase:barstar relative binding free energies $\left(\Delta \Delta G_{\text{binding}}s\right)$. We ran 10 ns/replica AREX simulations for the 14 mutations in the barnase:barstar mutation set in both the forward and reverse directions, resulting in a total of 28 $\Delta \Delta G_{\text{binding}}$ predictions. We first compared the predicted versus experimental $\Delta \Delta G_{\text{binding}}s$ and considered a mutation to be significantly discrepant if the 95% CIs of its predicted and experimental $\Delta \Delta G_{\text{binding}}s$ were not within 1 kcal/mol of each other. We observed relatively poor agreement (RMSE: 2.49, 95% CI: [1.32, 3.74] kcal/mol) with 7% (2/28) of the predictions having the wrong sign and 21% (6/28) of the predictions considered significantly discrepant (Figure 6B, Supplementary Figure 6A). Moreover, when we compared the forward and negative of the reverse $\Delta \Delta G_{\text{binding}}s$ for each mutation, we found that 21% (3/14) of mutations have poor internal consistency (i.e., the 95% CIs of the forward and negative reverse $\Delta \Delta G_{\text{binding}}s$ are not within 1 kcal/mol of each other) (Figure 6B). We refer to the following mutations, all with poor accuracy with respect to experiment, as significantly discrepant mutations: A42T, R87A, D35A, H102A, A29Y, Q83R (Supplementary Figure 6A). A subset of these mutations (A42T, R87A, A29Y, Q83R) also has poor internal consistency.

We next demonstrate that all mutations with significant discrepancy have sufficiently overlapping alchemical states, but some have slow $\Delta G_{complex}$ convergence. To assess state overlap, we checked for sufficient replica mixing in both phases of simulation and found that the replicas mix well, indicating that the $\Delta \Delta G_{\text{binding}}$ discrepancies are not a result of poor overlap of alchemical states (Supplementary Figure 5). We next determined whether the free energy difference of mutating WT→Mutant in each phase (ΔG) has converged by checking whether the slope of the last 5 ns of the ΔG time series (i.e., ΔG as a function of simulation time) is within statistical uncertainty of zero (0 ± 0.1 kcal/mol/ns). We found that 67% (4/6) of the significantly discrepant mutations (A42T, R87A, H102A, and Q83R) have $\Delta G_{complex}s$ with slow convergence, suggesting that the corresponding simulations may contain significant sampling problems (Figure 3E–G). For the remaining 33% (2/6) of significantly discrepant mutations (D35A and A29Y), the $\Delta G_{complex}s$ do converge within 10 ns (Figure 3G), which indicates that they may have minimal sampling problems, though it is possible that the slowest degrees of freedom in these simulations have correlation times longer than 10 ns and therefore have not yet been sampled.

Finally, we show that sampling problems often occur in complex phase simulations, especially for charge-changing mutations. We extended this analysis to all 28 barnase:barstar mutations and observed that most of the mutations (27/28) have ΔG_apo_s that converge within 10 ns/replica (Figure 3H). However, 25% (7/28) of mutations have $\Delta G_{complex}s$ that do not converge, some of which are mutations that have predicted $\Delta \Delta G_{\text{binding}}s$ close to experiment (R83Q, A39D, A35D) and should therefore be considered problematic mutations (Figure 3G). More importantly, this analysis indicates that convergence may be more difficult to achieve in the complex phase simulations, likely because of difficulties in sampling. Furthermore, out of the seven mutations with poor convergence, six of the mutations are charge-changing, suggesting that sampling may be more challenging for charge-changing mutations (Figure 3G).

In summary, we identified several barnase:barstar mutations with predicted $\Delta \Delta G_{\text{binding}}s$ that exhibit poor accuracy and described an approach for identifying mutations that potentially have significant sampling challenges. We found that 32% (9/28) of the mutations have discrepant $\Delta \Delta G_{\text{binding}}s$ or slow $\Delta G_{complex}$ convergence with 10 ns/replica AREX simulations. Moreover, while sampling problems are absent for terminally-blocked amino acid mutations, they are likely present in the complex phase for several barnase:barstar mutations, most of which involve charge-changes. In the next section, we attempt to identify the slow degrees of freedom causing sampling challenges.

### 2 Poor complex phase sampling can occur due to mutation-dependent slow protein or water degrees of freedom

We chose two significantly discrepant mutations for deeper analysis of potential sampling challenges, A42T and R87A, each of which also has poor internal consistency (Supplementary Figure 6A, Figure 3B). These mutations encompass distinct types of transformations: A42T is a reverse mutation that involves a neutral, small to medium amino acid change (ALA to THR) and R87A is a forward mutation that involves a charge-changing, large to small transformation (ARG to ALA). Both mutations have slowly converging complex phase free energy differences $\left(\Delta G_{complex}s\right)$ which are likely a result of sampling problems (Figure 3F). In this section, we confirm the presence of sampling problems in A42T and R87A complex phase simulations and identify slow degrees of freedom likely causing poor sampling.

#### 2.1 Sampling challenges can be caused by hindered protein conformational dynamics

**Figure 4.**
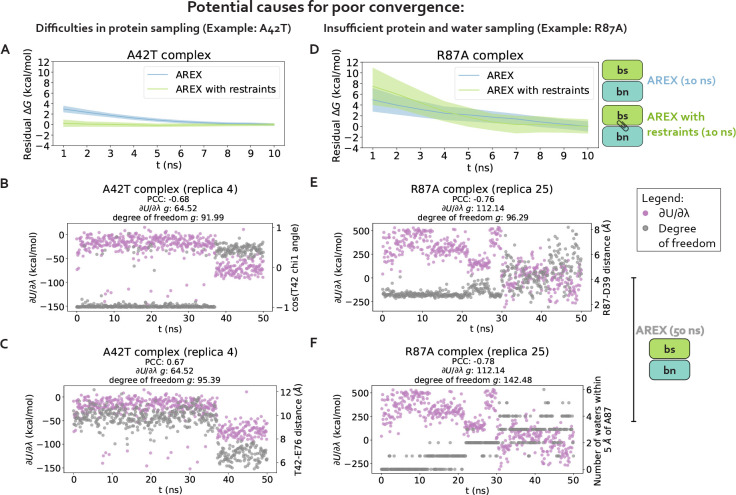
Complex phase convergence problems can arise due to insufficient sampling of protein and water degrees of freedom, e.g., a sidechain rotamer and intra-barstar contact for A42T and an interface contact and neighboring waters for R87A. **(A)** Residual complex phase free energy difference (ΔG) time series for AREX simulations of A42T (number of states = 24 and simulation time = 10 ns/replica), where the residual ΔG is computed as ΔG(*t*) − ΔG(*t* = 10ns). Blue curve represents the time series for the AREX simulation without restraints and green curve represents the time series for the AREX simulation with heavy atoms restraints (force constant = 50 kcal/molÅ^2^). Shaded regions represent ± two standard errors, which were computed by bootstrapping the decorrelated reduced potential matrices 200 times. **(B)** Time series for *∂U*/*∂λ* (left y-axis, purple) and *χ*_1_ angle for residue T42 (right y-axis, gray) for a representative replica (replica 4) of the A42T complex phase AREX simulation (number of states = 24, simulation time = 50 ns/replica). PCC indicates Pearson correlation coefficient and *g* indicates statistical inefficiency. **(C)** Time series for *∂U*/*∂λ* (left y-axis, purple) and T42-E76 distance (right y-axis, gray) for a representative replica (replica 4) of the A42T complex phase AREX simulation (number of states = 24, simulation time = 50 ns/replica). **(D)** Same as (A), but for R87A instead of A42T (number of states = 36 and simulation time = 10 ns/replica). **(E)** Time series for *∂U*/*∂λ* (left y-axis, purple) and R87-D39 distance (right y-axis, gray) for a representative replica (replica 25) of the R87A complex phase AREX simulation (number of states = 36, simulation time = 50 ns/replica). **(F)** Time series for *∂U*/*∂λ* (left y-axis, purple) and number of waters within 5 Å of A87 (right y-axis, gray) for a representative replica (replica 25) of the R87A complex phase AREX simulation (number of states = 36, simulation time = 50 ns/replica).

We first hypothesized that slow $\Delta G_{complex}$ convergence can be attributed to poor sampling of slow protein backbone or side chain motion in the A42T and R87A complex phase simulations. To test this hypothesis, we ran 10 ns/replica complex phase AREX simulations where we imposed restraints on the heavy-atom coordinates to eliminate protein motion as a source of slow degrees of freedom, and compared the $\Delta G_{complex}$ time series with and without restraints for each mutation. Because these restraints significantly reduce protein motion, if the slow $\Delta G_{complex}$ convergence is caused by slow protein motions that are insufficiently sampled, the restraints should eliminate the sampling problem and the $\Delta G_{complex}$ time series should converge immediately. The A42T $\Delta G_{complex}$ time series with restraints converges rapidly within 10 ns, lacking the downward trend that is present in the $\Delta G_{complex}$ time series without restraints and indicating that the A42T complex phase simulation has a protein sampling problem (Figure 4A). However, for R87A, the $\Delta G_{complex}$ time series with restraints is within error of the time series without restraints, exhibiting the same downward trend as the unrestrained time series, which suggests the lack of convergence in the R87A $\Delta G_{complex}$ is not solely caused by protein sampling problems (Figure 4D). Although this analysis can help determine the presence (or absence) of protein sampling problems, it does not identify the specific slow degrees of freedom that are likely causing sampling problems.

#### 2.2 Poor sampling can specifically be attributed to individual sidechain torsions, interfacial contacts, or nearby waters

We next determined the specific degrees of freedom which may be responsible for slow $\Delta G_{complex}$ convergence by identifying conformational degrees of freedom that are tightly coupled to *∂U*/*∂λ*. We are particularly interested in mutations with slowly-varying *∂U*/*∂λ* (i.e., highly correlated and with large statistical inefficiency, *g*), because slowly-varying *∂U*/*∂λ*s indicate slow $\Delta G_{complex}$ convergence. We monitored hundreds of protein and water degrees of freedom near the protein:protein interface over time because we observed that slow convergence is more common for $\Delta G_{complex}$ than ΔG_apo_ (Figure 3G–H). Specifically, we monitored the following degrees of freedom: backbone or rotameric torsion transitions near the interface, residue contacts within or between binding partners, and waters near the alchemical residue. We then computed the Pearson correlation coefficient (PCC) between *∂U*/*∂λ* and each degree of freedom, averaging over all replicas. For mutations with slowly-varying *∂U*/*∂λ*, the most highly coupled (largest magnitude PCC) degree of freedom is likely implicated in slow $\Delta G_{complex}$ convergence. We emphasize that the slow degrees of freedom discussed hereafter are relatively slow (in the context of the degrees of freedom we analyzed and in the timescales of our simulations), and that they are not necessarily the globally slowest degrees of freedom for each alchemical transformation.

**Figure 5.**
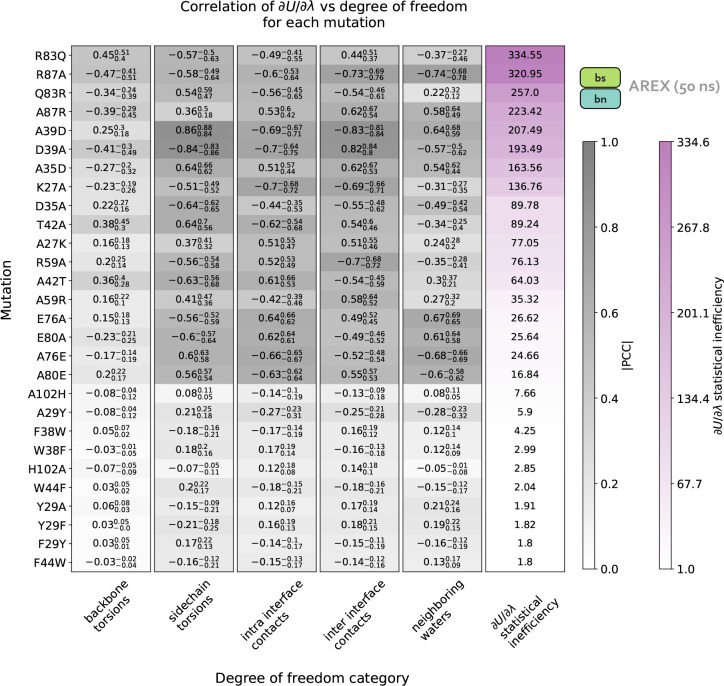
Charge-changing mutations demonstrate worse complex phase sampling than neutral mutations and the slowest degrees of freedom responsible for poor sampling are highly variable depending on the mutation. Data in this plot was generated from 50 ns/replica complex phase AREX simulations. Each row of the heatmap corresponds to a mutation and each of the first five columns corresponds to a degree of freedom category: backbone torsions, sidechain torsions, intra-interface contacts, inter-interface contacts, and neighboring waters. Each category contains a set of degrees of freedom, i.e., the backbone torsions category contains the *ϕ* and *ψ* angles for all interface residues, sidechain torsions contains the *χ*_1_, *χ*_2_, *χ*_3_, and *χ*_4_ angles for all interface residues (if the angle is present for the residue), intra-interface contacts contains pairs of interface residues that are within the same chain, inter-interface contacts contains pairs of interface residues that span different chains, and neighboring waters involves monitoring the number of waters within 5 Å of the mutating residue. Each heatmap value (in the first five columns) is the maximum of (the absolute value of) the Pearson correlation coefficients (PCCs) between *∂U*/*∂λ* and each of the degrees of freedom in the corresponding category for the corresponding mutation. For example, the top left value of the heatmap indicates that for R83Q, the backbone torsion with maximum correlation to *∂U*/*∂λ* has a PCC of 0.44. The background colors for the PCC values are different shades of gray, with darker grays indicating values closer to 1. The subscript and superscript values associated with each PCC represent the 95% confidence interval. Each heatmap value in the last column corresponds to the statistical inefficiency of *∂U*/*∂λ* across all replica trajectories for the corresponding mutation. Statistical inefficiency values are colored different shades of purple, with darker colors indicating larger values. The rows of the heatmap are ordered from highest to lowest by the statistical inefficiency of *∂U*/*∂λ* across all replicas. See **Detailed Methods** for more information about this analysis

For A42T, which has slowly-varying *∂U*/*∂λ* (*g* = 64.03, Figure 5), the degrees of freedom with the largest magnitude PCCs are the *χ*_1_ angle of T42 (PCC: −0.63, 95% CI: [−0.68, −0.56], Figure 5) and the distance between interface barstar residues T42 and E76 (PCC: 0.61, 95% CI: [0.53, 0.66], Figure 5). The time series of a typical replica show that both degrees of freedom are highly correlated with *∂U*/*∂λ* and slowly sample two metastable states during the 50 ns replica trajectory (Figure 4B–C). The relatively slow sampling of sidechain rotamer and interface contact metastable states (correlation time: 9.2 ns for T42 *χ*_1_ and 9.5 ns for T42-E76, Figure 4B–C) likely explains the slow convergence of the A42T $\Delta G_{complex}$ time series in Figure 3F. Water sampling does not seem to play a significant role in causing poor $\Delta G_{complex}$ convergence for A42T, as the waters near T42 are only weakly correlated to *∂U*/*∂λ* (PCC: 0.30, 95% CI: [0.21, 0.37], Figure 5).

For R87A, which also has slowly-varying *∂U*/*∂λ* (*g* = 320.95, Figure 5), the degree of freedom with the largest magnitude PCC is the number of waters near A87 (PCC −0.74, 95% CI: [−0.78, −0.68], Figure 5). The correlation is also particularly high for the distance between interface residues R87 (barnase) and D39 (barstar) (PCC: −0.73, 95% CI: [−0.76, −0.69], Figure 5). The time series of a representative replica shows that both degrees of freedom are highly correlated with *∂U*/*∂λ* (Figure 4F, E). Both degrees of freedom also have long correlation times (9.63 ns for R87-D39 and 14.2 ns for neighboring waters) and slow burn-in times, which suggests the slow convergence of R87A $\Delta G_{complex}$ is likely explained by slowness in R87-D39 and nearby waters (Figure 4E–F).

We next attempted to identify general trends in the slow degrees of freedom across all barnase:barstar mutations and found that there is no common degree of freedom (or category of degrees of freedom) that is implicated in all complex phase sampling problems (Figure 5). We observed that backbone torsions consistently show PCCs less than 0.5 in magnitude (which is smaller than the PCCs of the other degrees of freedom), indicating that backbone torsions are unlikely to be the primary cause of sampling problems. The other four categories—sidechain torsions, intra interface contacts, inter interface contacts, and neighboring waters—each have many high correlation values (magnitude of PCC greater than 0.5), but no single category explains the majority of sampling problems. Therefore, the slowest degrees of freedom are highly variable depending on the mutation (Figure 5).

Finally, we observed that for complex phase simulations, mutations involving charge-changes show slower convergence than charge-preserving transformations. 83% (10/12) of neutral mutations have *∂U*/*∂λ* time series with *g* < 10, whereas 100% (16/16) of charge-changing mutations have *g* > 10 (Figure 5). We emphasize that the slow convergence of charge-changing mutations only occurs in the complex (and not apo) phase (Figure 3H), indicating that introduction of a counterion to accommodate charge-changes does not significantly contribute to slow convergence. Instead, the sampling difficulties for charge-changing mutations likely emerge as a result of the strong network of electrostatic interactions at the barnase:barstar interface (Figure 1C).

One limitation of this work is that we studied only one protein:protein complex, and it is possible that other types of sampling problems are present in other protein:protein complexes. From our focused experiments, we cannot extrapolate how common the barnase:barstar sampling issues are for other protein:protein complexes, though it seems likely that the issues observed here are sufficiently fundamental in origin to be present in other complexes. It is worth remarking that the uniquely strong electrostatic nature of the barnase:barstar interface may exacerbate sampling challenges compared to other PPIs with less electrostatically driven binding. The barnase:barstar interface involves 14 hydrogen bonds, more than the average protein:protein complex [69]. Of the 14 hydrogen bonds, most involve at least one charged residue, which is also atypical for protein:protein complexes [69]. Further work will be necessary to determine the extent to which the sampling problems observed in barnase:barstar are similar to those in other protein:protein systems and identify other mechanisms by which sampling problems could manifest.

Another caveat of this work is that the degrees of freedom explored in this analysis are not exhaustive; other, more complex collective variables (e.g., identified by time-lagged independent component analysis (TICA) [75, 76]) may correlate with *∂U*/*∂λ* even more highly than those explored here. Nevertheless, our scan of simple degrees of freedom reveals specific slow degrees of freedom (sidechain torsions, interfacial contacts, or nearby waters) likely implicated in slow $\Delta G_{complex}$ convergence. Moreover, we found that the degrees of freedom causing poor sampling are highly dependent on the mutation. This analysis serves as an example approach for diagnosing sampling problems in other protein:protein complexes. In the next section, we explore approaches for ameliorating the sampling challenges.

### 3 Given sufficient simulation time, AREX and AREST can provide converged and accurate $\Delta \Delta G_{\text{binding}}$ predictions

We explored two potential solutions for overcoming the observed sampling challenges: (1) running much longer simulations with the same sampling strategy (AREX) with the goal of exceeding the relevant slow correlation times to enable convergence, and (2) using an enhanced sampling strategy that aims to reduce the correlation times to shorter timescales. For (2), we consider the addition of solute tempering to alchemical replica exchange (AREST) with the goal of significantly reducing correlation times at intermediate alchemical states. We explore the extent to which each approach improves convergence for the complex phase simulations of all barnase:barstar mutations, with a special focus on A42T and R87A.

#### 3.1 Significantly longer (50 ns/replica) complex phase AREX simulations yield improved $\Delta G_{complex}$ convergence and adequate sampling of slow conformational degrees of freedom

**Figure 6.**
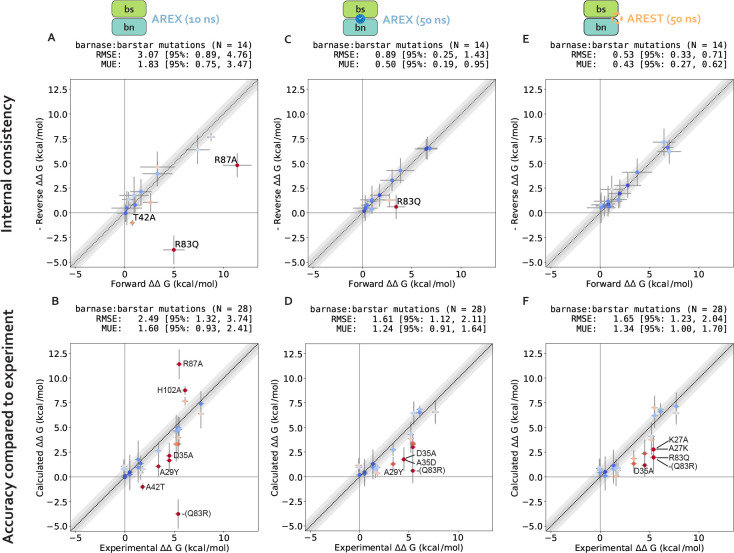
Running long (50 ns/replica) simulations of alchemical replica exchange (AREX) and alchemical replica exchange with solute tempering (AREST) yields improved $\Delta \Delta G_{\text{binding}}$ predictions with respect to 10 ns/replica AREX simulations. **(A)** Forward versus (negative of the) reverse $\Delta \Delta G_{\text{binding}}s$ for each barnase:barstar mutation computed from AREX simulations (number of states = 24 and 36 for neutral and charge mutations, respectively and simulation time = 10 ns/replica for each phase). The y = x (black dotted) line represents zero discrepancy between forward and (negative of the) reverse $\Delta \Delta G_{\text{binding}}$, the dark gray shaded region represents 0.5 kcal/mol discrepancy, and the light gray region represents 1 kcal/mol discrepancy. Data points are colored by how far they are from zero discrepancy (dark blue and red indicate close to and far from zero, respectively). Data points are labeled if the 95% CIs of the forward and (negative of the) reverse $\Delta \Delta G_{\text{binding}}s$ are not within 1 kcal/mol of each other. Error bars represent two standard errors and were computed by bootstrapping the decorrelated reduced potential matrices 200 times. Root mean square error (RMSE) and mean unsigned error (MUE) are shown with 95% confidence intervals obtained from bootstrapping the data 1000 times. **(B)** Calculated versus experimental $\Delta \Delta G_{\text{binding}}$ for each barnase:barstar mutation computed from AREX simulations (number of states = 24 and 36 for neutral and charge mutations, respectively and simulation time = 10 ns/replica for each phase). The y = x (black dotted) line represents zero discrepancy between calculated and experimental $\Delta \Delta G_{\text{binding}}s$, the dark gray shaded region represents 0.5 kcal/mol discrepancy, and the light gray region represents 1 kcal/mol discrepancy. Data points are labeled if the 95% CIs of the calculated and experimental $\Delta \Delta G_{\text{binding}}s$ are not within 1 kcal/mol of each other. For more details on the plot and error bars, refer to the caption for panel A. **(C)** Same as (A), but using 50 ns/replica AREX simulations for the complex phase and 10 ns/replica AREX simulations for the apo phase instead of 10 ns/replica AREX simulations for both phases. **(D)** Same as (B), but using 50 ns/replica AREX simulations for the complex phase and 10 ns/replica AREX simulations for the apo phase instead of 10 ns/replica AREX simulations for both phases. **(E)** Same as (A), but using 50 ns/replica AREST simulations for the complex phase and 10 ns/replica AREX simulations for the apo phase instead of 10 ns/replica AREX simulations for both phases. **(F)** Same as (B), but using 50 ns/replica AREST simulations for the complex phase and 10 ns/replica AREX simulations for the apo phase instead of 10 ns/replica AREX simulations for both phases.

We first demonstrate that running longer (50 ns/replica) complex phase AREX simulations improves barnase:barstar $\Delta \Delta G_{\text{binding}}$ predictions. We found that the accuracy of the predictions improved: the RMSE decreased from 2.49 (95% CI: [1.32, 3.74]) kcal/mol with 10 ns/replica AREX to 1.61 (95% CI: [1.12, 2.11]) kcal/mol with 50 ns/replica AREX (Figure 6D). Moreover, with 50 ns/replica AREX simulations, 86% (24/28) of predictions are close to experiment and all mutations have the correct sign (Figure 6D, Supplementary Figure 6B). We also found that the internal consistency significantly improved: the RMSE decreased from 3.07 (95% CI: [0.89, 4.76]) kcal/mol for 10 ns/replica AREX to 0.89 (95% CI: [0.25, 1.43]) kcal/mol for 50 ns/replica AREX (Figure 6C). Finally, we found that with 50 ns/replica AREX simulations, the convergence of $\Delta G_{complex}$ improved significantly, such that 100% (28/28) of mutations have converged (Supplementary Figure 9A). We then confirmed that the improved convergence for A42T and R87A is a result of more thoroughly sampling of the likely slowest degrees of freedom associated with each mutation. Examination of representative time series shows that between 10–50 ns, the slow degrees of freedom are sampled more comprehensively than with only 10 ns (Figure 4B, F), indicating that the correlation times of the slow degrees of freedom for A42T and R87A are on the order of 10 ns (rather than much longer).

We next show that the poor accuracy with respect to experiment for mutations with significantly discrepant $\Delta \Delta G_{\text{binding}}s$ (even after 50 ns) are likely due to errors in force field parameters or extreme sampling problems. Despite the improved predictions obtained from running longer AREX, 14% (4/28) of the mutations still demonstrate significantly poor accuracy: D35A, A35D, Q83R, and A29Y (Figure 6C). Common reasons for discrepant $\Delta \Delta G_{\text{binding}}$ predictions include insufficient protein or water sampling, errors in force field parameters, and failure to model multiple protonation states [32]. We found that A29Y has a significantly discrepant $\Delta \Delta G_{\text{binding}}$ (and relatively poor internal consistency) because the mutant tyrosine residue does not sample the relevant energetically favorable orientations that enable it to contribute favorably to the barnase:barstar interface (details in Supplementary Information).

We next investigated the causes of discrepancy for the remaining significantly discrepant mutations (D35A, A35D, and Q83R), all of which pass the internal consistency check with sufficient simulation time (100 ns/replica for Q83R and 50 ns/replica for the other two mutations, see Figure 6C and Supplementary Table 1). We first assessed whether the discrepancies are a result of failing to account for all relevant protonation states and found that protonation states are not the cause of these discrepancies (see Supplementary Information). Given the absence of protonation state problems, the discrepancies are likely due to inaccurate force field parameters or insufficient sampling (of a slow degree of freedom with a correlation time longer than 50 ns). However, it is worth noting that with sufficient simulation time, the sign is correct for each of these discrepant $\Delta \Delta G_{\text{binding}}s$, indicating that the estimates are still useful in characterizing whether a mutation is energetically favorable or unfavorable (Figure 6D).

**Figure 7.**
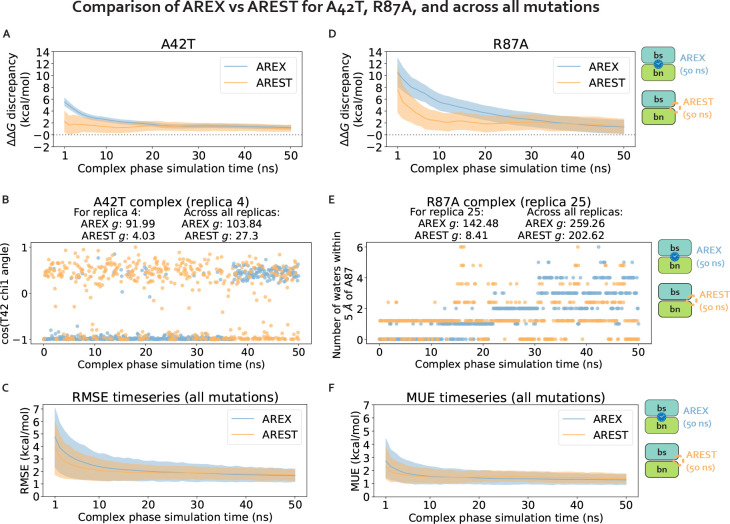
Alchemical replica exchange with solute tempering (AREST) and alchemical replica exchange (AREX) demonstrate comparable convergence for most barnase:barstar mutations. **(A)** $\Delta \Delta G_{\text{binding}}$ discrepancy (with respect to experiment) time series for A42T. The discrepancy was computed as ${\Delta G}_{\text{complex}}-{\Delta G}_{\text{apo}}-{\Delta \Delta G}_{\text{experiment}}$, where $\Delta G_{complex}$ corresponds to the (AREX or AREST) complex phase ΔG at a particular time point, ${\Delta G}_{\text{apo}}$ corresponds to the apo phase ΔG computed from a 10 ns/replica AREX simulation, and ${\Delta \Delta G}_{\text{experiment}}$ is the experimental value from Schreiber et al [67]. AREX time series shown in blue and AREST time series (with radius = 0.5 nm, $T_{\text{max}}=600K)$ shown in orange. Number of states is 24 for both AREX and AREST. Shaded regions represent ± two standard errors and and were computed by bootstrapping the decorrelated reduced potential matrices 200 times. Gray dashed line indicates $\Delta \Delta G_{\text{binding}}$ discrepancy = 0. **(B)** Time series of the *χ*_1_ angle for residue T42 for a representative replica (replica 4) of the A42T complex phase AREX simulation (blue) and A42T complex phase AREST simulation (orange) (number of states = 24, simulation time = 50 ns/replica). *g* indicates statistical inefficiency. **(C)** Time series of the root mean square error (RMSE) (with respect to experiment) for the $\Delta \Delta G_{\text{binding}}s$ of all barnase:barstar mutations. The $\Delta \Delta G_{\text{binding}}s$ used to compute the RMSE at each time point were computed as ${\Delta G}_{\text{complex}}-{\Delta G}_{\text{apo}}$ for each mutation, where $\Delta G_{complex}$ corresponds to the (AREX or AREST) complex phase ΔG at a particular time point and ${\Delta G}_{\text{apo}}$ corresponds to the apo phase ΔG computed from a 10 ns/replica AREX simulation. AREX time series shown in blue and AREST time series (with radius = 0.5 nm, $T_{\text{max}}=600K)$ shown in orange. Number of states is 24 for neutral mutations and 36 for charge-changing mutations. Shaded regions represent ± two standard errors and were computed by bootstrapping 1000 times. **(D)** Same as (A), but for R87A instead of A42T. Number of states is 36 for both AREX and AREST. **(E)** Time series of the number of waters within 5 Å of residue A87 for representative replica (replica 25) of the R87A complex phase AREX simulation (blue) and R87A complex phase AREST simulation (orange) (number of states = 36, simulation time = 50 ns/replica). **(F)** Same as (C) but for mean unsigned error (MUE) instead of RMSE.

Despite the exceptions described above, we emphasize that running longer AREX significantly improved our barnase:barstar predictions (RMSE is 1.61, 95% CI: [1.12, 2.11] kcal/mol, Figure 6D), indicating that for several mutations, sampling was insufficient with 10 ns/replica AREX but sufficient with 50 ns/replica. Moreover, 50 ns/replica may not be necessary depending on the desired accuracy, e.g., to achieve an RMSE of less than 2 kcal/mol for barnase:barstar predictions, ~20 ns/replica AREX simulations should be sufficient (Figure 7C).

#### 3.2 AREST convergence is comparable to that of AREX for most mutations

We next demonstrate that running 50 ns/replica AREST simulations also yields improved barnase:barstar $\Delta \Delta G_{\text{binding}}$ (compared to 10 ns/replica AREX simulations). We ran 50 ns/replica AREST (with radius = 0.5 nm and *T*_max_ = 600 K, see Supplementary-Information for details on REST parameter selection) for the complex phase of all barnase:barstar mutations and observed sufficient replica mixing for all mutations (Supplementary Figure 10). We observed significant improvement in the accuracy with respect to experiment; the RMSE decreased from 2.49 (95% CI: [1.32, 3.74]) kcal/mol for 10 ns/replica AREX to 1.65 (95% CI: [1.23, 2.04]) kcal/mol for 50 ns/replica AREST (Figure 6F, Supplementary Figure 6C). We also found that the internal consistency significantly improved with the RMSE decreasing from 3.07 (95% CI: [0.89, 4.76]) kcal/mol for 10 ns/replica AREX to 0.53 (95% CI: [0.33, 0.71]) kcal/mol for 50 ns/replica AREST (Figure 6E). Finally, we also observed that with 50 ns/replica AREST simulations, 100% (28/28) of the complex free energy differences $\left(\Delta G_{complex}s\right)$ converge (Supplementary Figure 9B).

We next show that AREST converges more efficiently than AREX for two mutations with sampling problems, but not for the rest of the barnase:barstar mutations. We monitored the discrepancy in predicted $\Delta \Delta G_{\text{binding}}$ with respect to experiment as a function of time and compared the discrepancy time series for 50 ns/replica AREST versus that from 50 ns/replica AREX simulations. We analyzed the discrepancies in $\Delta \Delta G_{\text{binding}}s$ for each of the seven mutations identified as potentially containing sampling problems due to slow $\Delta G_{complex}$ convergence with 10 ns/replica AREX: A42T, R87A, R83Q, Q83R, H102A, A35D, and A39D (Figure 3G). For A42T, the AREST discrepancy flattens out (to a close-to-zero discrepancy) more quickly than that of AREX, indicating that for A42T, AREST converges with less simulation time than AREX (Figure 7A). Similarly, for R87A, the AREST discrepancy starts to flatten out around 10 ns, while the AREX discrepancy doesn’t start to flatten out until ~40 ns, demonstrating that for R87A, AREST converges faster than AREX (Figure 7D). We next investigated why AREST yields faster convergence by comparing AREX and AREST sampling of the likely slowest degrees of freedom (T42 *χ*_1_ angle for A42T and number of waters near A87 for R87A) in representative time series. We found that AREST more thoroughly samples these degrees of freedom and the statistical inefficiencies of the AREST time series are smaller than those of AREX, indicating that the faster convergence of AREST is due to reduction of relevant correlation times (Figure 7B, E).

Importantly, we found that for the remaining 71% (5/7) of mutations potentially containing sampling problems, the discrepancy in $\Delta \Delta G_{\text{binding}}$ does not converge to zero significantly faster for AREST than AREX (Supplementary Figure 11). Finally, to assess convergence across all mutations, we monitored the root mean square error (RMSE) and mean unsigned error (MUE) over time and observed that for both RMSE and MUE, the AREX and AREST time series are within error of each other (Figure 7C, F). Therefore, although AREST shows better convergence than AREX for A42T and R87A, AREST convergence is comparable to that of AREX when comparing the two sampling strategies over all barnase:barstar mutations.

## DISCUSSION

### Widespread application of RBFE calculations to protein:protein complexes is primarily limited by the simulation time required to achieve reliable estimates

For some mutations, running RBFE calculations long enough to achieve converged, accurate, and reliable predictions can be computationally expensive, depending on the simulation time required and the computing resources available. For example, to achieve highly accurate RBFE predictions (RMSE ~1.6 kcal/mol) for barnase:barstar, the most challenging mutations (i.e., charge-changing mutations with sampling challenges) require 50 ns/replica for the complex phase and 10 ns/replica for the apo phase. This amounts to ~220 graphics processing unit (GPU) hours per mutation on an NVIDIA A100 graphics card—at the cost of roughly $920 per mutation on an equivalent instance on Amazon Web Services (AWS) (Supplementary Information A.12). However, we emphasize that we obtained converged and accurate $\Delta \Delta G_{\text{binding}}$ estimates for most of the mutations with 10 ns/replica AREX (Section 1.2), indicating that most mutations would not require such computationally expensive simulations (and instead would cost ~62 GPU hours and $260 per mutation on AWS). Taken together, our results demonstrate that given current best practices sampling strategies and state-of-the-art computing resources, the primary limiting factor in applying RBFE calculations to protein:protein complexes is the computational cost associated with achieving sufficient sampling for a small subset of mutations.

Given that similar types of sampling problems are also challenging for small molecule transformations [48, 49, 77], finding ways to reduce computational cost for alchemical transformations with difficult sampling problems will be highly useful for the development of alchemical free energy calculations in general. One straightforward approach for reducing computational cost involves waiting for improvements in hardware. GPU performance has rapidly improved over the last decade and will continue to improve in the coming years [78]. There are also particularly exciting developments in the realm of cheaper parallelization through the introduction of wider GPUs that enable a single GPU to be partitioned into multiple instances (e.g., NVIDIA’s Multi-Instance GPU feature).

### Improvement of AREX and AREST simulation parameters may reduce the simulation time required for converged ΔΔG estimates for mutations with sampling challenges

Beyond anticipating advancements in hardware, a promising avenue for decreasing computational cost involves further optimizing the AREX and AREST simulation parameters used in this study. For both AREX and AREST, we chose the same number of alchemical intermediate states for all neutral mutations and a different, larger number of states for all charge-changing mutations. Additionally, we defined the alchemical and REST scaling protocols for each state according to simple, piecewise linear functions. Moreover, for AREST, we chose the REST parameters (radius and *T*_max_) by exploring a small set of extreme REST parameters (Supplementary Information D).

Although we confirmed that our AREX and AREST parameter choices do not result in any replica mixing bottlenecks (Supplementary Figures 2, 5, 10), there are likely alternative protocol parameters which could provide more efficient $\Delta \Delta G_{\text{binding}}$ convergence. Ideally, each mutation would have optimized protocol parameters that provides a converged and accurate $\Delta \Delta G_{\text{binding}}$ estimate in the minimal amount of simulation time. However, because the search space for each of the parameters is large, brute-force optimization is unfeasible and even exploration of extreme values for each parameter for each mutation would be quite computationally expensive. Therefore, future work could involve development of methods for mutation-specific parameter optimization. Furthermore, there are also opportunities for optimizing mutation-independent protocol parameters, such as the integrator timestep [79], alchemical functional form [80–83], and Particle Mesh Ewald error tolerance [61] which may reduce simulation time.

### Adaptation of other enhanced sampling methods for use in alchemical free energy calculations may also decrease the simulation time required to sufficiently sample difficult transformations

There are many existing methods for enhancing sampling in molecular dynamics simulations [84], many of which accelerate sampling of known slow degrees of freedom in a targeted manner [44–47, 85–90]. Some existing enhanced sampling methods also identify the slow degrees of freedom (as an intermediate step) [91, 92], but they do not necessarily identify the slow degrees of freedom that are most highly coupled to the alchemical coordinate (i.e. *∂U*∕*∂λ*), which are are responsible for slow convergence of RBFEs. Future work could involve incorporating existing enhanced sampling methods into alchemical free energy calculations to further improve sampling and convergence, as has been demonstrated for simple test systems [93]. Furthermore, when adapting methods that identify slow degrees of freedom, it will be important to account for coupling to the alchemical coordinate.

## CONCLUSIONS

In this work, we explored the sampling challenges associated with applying relative binding free energy (RBFE) calculations to estimate the impact of protein mutations in a model protein:protein complex (barnase:barstar). We found that sampling problems are absent when the mutation is not located in the context of a complex network of protein interactions (i.e. in terminally-blocked amino acids), but are present in the complex phase for several barnase:barstar mutations, yielding slow convergence of $\Delta G_{complex}s$. Moreover, most of the mutations with complex phase sampling and convergence problems involve charge-changes. Furthermore, we attributed the barnase:barstar complex phase sampling problems to specific slow degrees of freedom (individual sidechain torsions, interfacial contacts, and nearby waters) which are highly dependent on the mutation. Finally, we found that given sufficient simulation time (50 ns/replica), both AREX and AREST can address most of the aforementioned sampling problems, with both methods demonstrating comparable convergence for most mutations. Therefore, for the barnase:barstar mutations in this study, AREST does not significantly reduce the simulation time required for converged ΔΔG estimates.

Ultimately, our analyses and findings provide a model framework for diagnosing and mitigating sampling problems in other protein:protein complexes. By facilitating deep investigation of these sampling challenges in an open-source manner, our study lays the groundwork for the development of better methods for improving sampling in protein:protein RBFE calculations and free energy calculations in general.

## Supplementary Material

Supplement 1
