# Supplementary material for: Identifying and overcoming the sampling challenges in relative binding free energy calculations of a model protein:protein complex
Source: bioRxiv. 2023 Mar 8:2023.03.07.530278. Preprint. [Version 1] doi: 10.1101/2023.03.07.530278 (PMC10028896; doi:10.1101/2023.03.07.530278)
Supplement: Supplementary file 1 [file NIHPP2023.03.07.530278v1-supplement-1.pdf]

bioRxiv preprint doi: https://doi.org/10.1101/2023.03.07.530278; this version posted March 8, 2023. The copyright holder for this preprint (which was not certified by peer review) is the author/funder, who has granted bioRxiv a license to display the preprint in perpetuity. It is made available under aCC-BY 4.0 International license.

Supplementary Information

Ivy Zhang (ORCID: 0000-0003-0628-6276)[1, 2], Dominic A. Rufa (ORCID: 0000-0003-0930-9445)[1, 3], Iván Pulido (ORCID: 0000-0002-7178-8136)[1], Michael M. Henry (ORCID: 0000-0002-3870-9993)[1], Laura E. Rosen (ORCID: 0000-0002-8030-0219)[4], Kevin Hauser (ORCID: 0000-0002-4579-4794)[4], Sukrit Singh (ORCID: 0000-0003-1914-4955)[1], John D. Chodera (ORCID: 0000-0003-0542-119X)[1]

[1]Computational and Systems Biology Program, Sloan Kettering Institute, Memorial Sloan Kettering Cancer Center, New York, NY 10065; [2]Tri-Institutional PhD Program in Computational Biology and Medicine, Weill Cornell Medical College, Cornell University, New York, NY 10065; [3]Tri-Institutional PhD Program in Chemical Biology, Weill Cornell Medical College, Cornell University, New York, NY 10065; [4]Vir Biotechnology, San Francisco, CA, USA

***For correspondence:**
john.chodera@choderalab.org (JDC)

A Detailed Methods

A.1 Code Availability

The Python code used to produce the results discussed in this paper is distributed open-source under a MIT license and is available at https://github.com/choderalab/perses-barnase-barstar-paper.

Core dependencies include Perses 0.10.1 [50], OpenMMTools 0.21.5 (https://github.com/choderalab/openmmtools), MDTraj 1.9.7 [94], and pymbar 3.1.1 [60]. OpenMM 8.0.0beta (https://anaconda.org/conda-forge/openmm/files?version=8.0.0beta — build 0), a development version of OpenMM 7 [26], was used to generate the input files for alchemical replica exchange (AREX) and alchemical replica exchange with solute tempering (AREST), run equilibration, and run AREX for the terminally-blocked amino acids. OpenMM 7.7.0.dev2 (https://anaconda.org/conda-forge/openmm/files?version=7.7.0dev2), a development version of OpenMM 7 [26] which was built after OpenMM 8.0.0beta and contains a performance enhancement for AREX and AREST, was used for running all other AREX and AREST simulations. ΔΔG comparison plots were generated with cinnabar 0.3.0 (https://github.com/OpenFreeEnergy/cinnabar). All other plots were generated using Matplotlib 3.5.2 [95].

A.2 Structure preparation

Capped peptides: To create structures for the terminally-blocked amino acids, tleap from AmberTools 21.9 [96] was used to generate the ACE-, NME-capped (ACE-X-NME) and zwitterionic ALA-capped (ALA-X-ALA) peptides in idealized alpha helical conformations (see https://github.com/choderalab/perses-barnase-barstar-paper/blob/main/input_files/generate_peptide_pdbs.py).

Barnase:barstar: To create a structural model of the wild-type (WT) barnase:barstar complex, chains A and D (which correspond to barnase and barstar, respectively) were extracted from the crystal structure with PDB ID 1BRS [67] because they are the chains with the highest overall quality (see wwPDB X-ray Structure Validation Report). Schrodinger Maestro 2021-2 [97] was used to prepare the structure with the Protein Prep Wizard, i.e., delete the other chains, fill in missing side chains and loops, cap the termini, add hydrogens, and optimize the hydrogen bond network (using pH 8.0, the pH used in Schreiber et al. binding experiments [67]). HIS18 (in barnase) was protonated as HID, HIS102 (in barnase) was protonated as HIE, and H17 (in barstar) was protonated as HID. Default settings were used unless otherwise noted. Because Maestro added NMA caps as inserted residues (i.e., the residue ID was the same as the preceding residue with the addition of an "A"), OpenMM 8.0.0beta [26] was used to rename the NMA residue to NME as well as renumber the NME residue and all subsequent residues to have residue IDs incremented by 1 (see https://github.com/choderalab/perses-barnase-barstar-paper/blob/main/input_files/renumber.py).

Although the experimental relative binding free energy ($\Delta\Delta G_{binding}$) data (Schreiber et al. [67]) was generated using the WT sequences of barnase and barstar, which contain cysteines at barstar residues 40 and 82,

bioRxiv preprint doi: https://doi.org/10.1101/2023.03.07.530278; this version posted March 8, 2023. The copyright holder for this preprint (which was not certified by peer review) is the author/funder, who has granted bioRxiv a license to display the preprint in perpetuity. It is made available under aCC-BY 4.0 International license.

the 1BRS structure contains alanines at those positions. We did not mutate residues 40 and 82 to cysteines in our structural model because it has been demonstrated that the structures, activities, and stabilities of mutant (A40 and A82) barstar are similar to those of WT (C40 and C82) barstar [98].

The prepared WT structure was used as a starting structure for forward mutations. For the reverse mutations, the starting structures were mutant barnase and barstar structures, which were generated by mutating the residue of interest in the prepared WT structure using Maestro 2021-3 [97]. The sidechain rotamer that best matched the sidechain orientation of the WT residue was selected.

For the D35A and K27A experiments (accounting for multiple protonation states), models of barstar with ASH35, barnase with LYN27, and terminally blocked amino acids with ASH or LYN were generated by modifying the protonation state of the prepped WT structures using OpenMM 8.0.0beta [26] (see https://github.com/choderalab/perses-barnase-barstar-paper/blob/main/input_files/generate_nonstandard_protonation_states.py).

A.3 System solvation and parameterization

Solvation and parameterization were performed with OpenMM 8.0.0beta [26]. The systems were solvated using the TIP3P rigid water model [99] in a cubic box with 12 Å and 17 Å solvent padding on all sides for barnase:barstar and terminally-blocked amino acids, respectively. The solvated systems were then minimally neutralized with 50 mM NaCl using the Li/Merz ion parameters of monovalent ions for the TIP3P water model (12-6 normal usage set) [100]. The systems were parameterized with the Amber ff14SB force field [101]. Amber ff14SB allows naked charges on certain hydrogens, i.e., atoms with a non-zero charge, but zero σ or ϵ. To prevent naked charges from causing simulation failures due to nuclear fusion when enhanced sampling strategies are employed, a small padding was added to each non-water atom with $\sigma = 0$ nm or $\epsilon = 0$ nm. If the atom had $\sigma = 0$ nm, 0.06 nm padding was added. If the atom had $\epsilon = 0$ kJ/mol, 0.0001 kJ/mol padding was added. Finally, if $\epsilon = 0$ kJ/mol and $\sigma = 1$, sigma was set to 0.1 nm. Full details and scripts can be found at: https://github.com/choderalab/perses-barnase-barstar-paper/blob/main/scripts/01_generate_solvated_inputs/generate_solvated_inputs.py.

A.4 System equilibration

To ensure that our experiments regarding convergence are not the result of structure preparation errors or crystallographic artifacts abruptly followed by production simulation, the barnase:barstar systems were gently equilibrated over 9 stages based on a previously described protocol [102] using OpenMM 8.0.0beta [26]. The number of steps, temperature, ensemble, collision rate, timestep, and force constant for each stage are detailed in the aforementioned reference. The reference protocol was run with a few adjustments:

1. The heavy atoms were restrained in the first four stages, backbone atoms were restrained in the next four stages, and no atoms were restrained for the last stage,
2. A Langevin integrator was used (see below for more details), so the Berendsen thermostat in the reference protocol was not necessary,
3. The last stage of gentle equilibration was extended to 9.25 ns (instead of 5 ns), so that the whole equilibration protocol would involve 10 ns of simulation.

The energy minimization stages were performed using the OpenMM 8.0.0beta LocalEnergyMinimizer with an energy tolerance of 10 kJ/mol. The molecular dynamics stages used the OpenMM 8.0.0beta LangevinMiddleIntegrator [79, 103, 104]. Hydrogen atom masses were set to 3 amu by transferring mass from connected heavy atoms, bonds to hydrogen were constrained, and center of mass motion was not removed. Pressure was controlled by a molecular-scaling Monte Carlo barostat with a pressure of 1 atmosphere, a temperature of 300 K, and an update interval of 50 steps. Non-bonded interactions were treated with the Particle Mesh Ewald method [105] using a real-space cutoff of 1.0 nm and an Ewald error tolerance of 0.00025, with grid spacing selected automatically. Long range anisotropic dispersion corrections were applied to un-scaled (non-REST and non-alchemical) steric interactions [106]. Because their structural models did not originate from crystal structures, the terminally-blocked amino acid systems were not equilibrated with the gentle equilibration protocol; they were minimized and then equilibrated for 10 ns without

bioRxiv preprint doi: https://doi.org/10.1101/2023.03.07.530278; this version posted March 8, 2023. The copyright holder for this preprint (which was not certified by peer review) is the author/funder, who has granted bioRxiv a license to display the preprint in perpetuity. It is made available under aCC-BY 4.0 International license.

restraints in the NPT ensemble at 300 K with a collision rate of 2 picoseconds^{-1} and a timestep of 2 femtoseconds. For the barnase:barstar complex systems, a virtual bond was added between the first atoms of each protein chain to ensure that the chains are imaged together. Default parameters were used unless noted otherwise. Further details on the equilibration protocol are available at: https://github.com/choderalab/perses-barnase-barstar-paper/blob/main/scripts/02_run_equilibration/run_equilibration.py.

A.5 Free energy calculation input file preparation

The hybrid topology, positions, and system for each transformation were generated using Perses 0.10.1 [50] and OpenMM 8.0.0beta [26]. The hybrid topology was generated using a single topology approach. The hybrid positions were assembled by copying the positions of all atoms in the WT ("old") topology and then copying the positions of the atoms unique to the mutant ("new") residue (i.e., unique new atoms). The unique new atom positions were generated using the Perses `FFAllAngleGeometryEngine`, which probabilistically proposes positions for one atom at a time based on valence energies alone. Further details on hybrid topology, positions, and system generation (including definitions of the valence, electrostatic, and steric energy functions) are available in the Perses `RESTCapableHybridTopologyFactory` class.

For charge-changing mutations, counterions were added to neutralize the mutant system by selecting water molecules in the WT system that are initially at least 8 Å from the solute and alchemically transforming the WT water molecules into sodium or chloride ions in the mutant system. For example, if the mutation was ALA→ASP, a water molecule in the WT ALA system was transformed into a sodium ion in the mutant ASP system to keep the system at the ASP endstate neutral. If the mutation was GLU→ALA, a water molecule in the WT GLU system was transformed into a chloride ion in the mutant ALA system. Additional details on the counterion implementation can be found in the Perses `_handle_charge_changes()` function found in `perses.app.relative_point_mutation_setup`.

To prevent singularities from arising when turning off the nonbonded interactions involving unique old or unique new atoms, a softcore approach was used that involves "lifting" unique old or unique new interaction distances into the "4th dimension." A padding distance ($w(\lambda)$, see equation 2) was added to the interaction distances involving unique old or unique new atoms so that the atoms could not be on top of each other [62]. w_{lifting} (the maximum value for $w(\lambda)$) was selected to be 0.3 nm and when AREX was performed for all terminally-blocked amino acid mutations, replica mixing was sufficient for all mutations, indicating that the thermodynamic length between alchemical states was reasonable even given the softcore lifting term (Supplementary Figure 2). Given that the 4D lifting approach involves lifting both the electrostatic and steric interactions, multi-stage alchemical protocols (where electrostatics must be turned off before sterics) are not necessary for scaling on or off the electrostatic and steric interactions. Therefore, a simple linear protocol was used for interpolating the valence, nonbonded, and lifting terms (Supplementary Figure 1A).

A.6 Alchemical replica exchange

Alchemical replica exchange (AREX) simulations were performed using Perses 0.10.1 [50], OpenMMTools 0.21.5 (https://github.com/choderalab/openmmtools). OpenMM 8.0.0beta [26] was used for the terminally-blocked amino acids and OpenMM 7.7.0.dev2 [26] was used otherwise. The alchemical protocol defined with evenly spaced λ values between 0 and 1. Before AREX was performed, the positions were minimized at each of the alchemical states using the OpenMM `LocalEnergyMinimizer` with an energy tolerance of 10 kJ/mol and a maximum of 100 iterations (except for D39A, A76E, and A39D complex phase AREST simulations, which were minimized without a limit on the number of iterations because instabilities were present with only 100 iterations). Each AREX cycle consisted of running 250 steps (4 femtosecond timestep) with the OpenMM 8.0.0beta `LangevinMiddleIntegrator` [79, 103, 104] at a temperature of 300 K, a collision rate of 1 picosecond^{-1}, and a constraint tolerance of 1e-6. All-to-all replica swaps were attempted every cycle [66]. Replica mixing plots were created using OpenMMTools 0.21.5 (https://github.com/choderalab/openmmtools) to extract the mixing statistics from the AREX trajectories. Default settings were used unless otherwise noted. For full details on the AREX implementation: https://github.com/choderalab/perses-barnase-barstar-paper/blob/main/scripts/04_run_repex/run_repex.py.

For terminally-blocked amino acid mutations, the two simulation phases involved different types of caps

bioRxiv preprint doi: https://doi.org/10.1101/2023.03.07.530278; this version posted March 8, 2023. The copyright holder for this preprint (which was not certified by peer review) is the author/funder, who has granted bioRxiv a license to display the preprint in perpetuity. It is made available under aCC-BY 4.0 International license.

— the first phase was ACE-X-NME and the other phase was ALA-X-ALA, where X is an amino acid. AREX simulations were run for each phase using 12 replicas for neutral mutations and 24 replicas for charge-changing mutations (including H102A, even though histidine was modeled as HIE). While ASH35A and LYN27A are both neutral mutations, 24 replicas were used for each to allow for direct comparison with D35A and K27A. Replicas mixed well for all mutations, indicating good phase space overlap (Supplementary Figure 2). 5000 cycles (i.e., 5 ns) were run for each replica, resulting in 60 ns of sampling per phase per neutral mutation and 120 ns of sampling per phase per charge-changing mutation.

For barnase:barstar mutations, apo and complex phase AREX simulations were performed with 24 replicas for neutral mutations and 36 replicas for charge-changing mutations (including H102A, even though histidine was modeled as HIE). While ASH35A and LYN27A are both neutral mutations, we used 36 replicas for each mutation to allow for direct comparison with D35A and K27A. Replicas mixed well for all mutations, indicating good phase space overlap (Supplementary Figure 5). 10000 cycles were initially run per replica (10 ns/replica), resulting in 240 ns of sampling phase per neutral mutation and 360 ns of sampling per phase per charge-changing mutation. The complex phase simulations were extended to 50 ns/replica, resulting in 1200 ns per phase per neutral mutation and 1800 ns per phase per charge-changing mutation.

To improve the accuracy of our predicted free energy differences, the sampled alchemical states were bookended with "virtual endstates," which were not sampled during free energy calculation, but for which reliable estimates of the physical endstates could be robustly produced during analysis. In these bookended endstates, nonbonded interaction energies were defined using the more accurate, but more computationally expensive, Lennard Jones with Particle Mesh Ewald (LJPME) method [107] to better account for the heterogeneous long-range dispersion interactions known to be important when creating or destroying many atoms in alchemical free energy calculations [106]. For full details on the unsampled endstate implementation, see: `perses.dispersed.utils.create_endstates_from_real_systems()`.

To run AREX simulations with heavy-atom coordinate restraints, an OpenMM 7.7.0.dev2 `CustomCVForce` was added to the hybrid system with the energy expression:

$$K_{\mathrm{RMSD}}\,(\mathrm{RMSD})^2 \tag{8}$$

where K_{RMSD} (the harmonic force constant) was chosen to be 50 kcal/molÅ^2 in order to sufficiently reduce heavy-atom motion without causing instabilities. RMSD was computed using an OpenMM 7.7.0.dev2 `RMSDForce` [26] (added as a collective variable to the `CustomCVForce`). The two forces (`CustomCVForce` and `RMSDForce`) enable restraint of heavy atoms to their initial positions. For full details on the restraint implementation: https://github.com/choderalab/perses-barnase-barstar-paper/blob/main/scripts/04_run_repex/run_repex.py.

A.7 Alchemical replica exchange with solute tempering (AREST)

The AREST simulations were performed using Perses 0.10.1 [50], OpenMMTools 0.21.5 (https://github.com/choderalab/openmmtools), and OpenMM 7.7.0.dev2 [26] with the same parameters used in alchemical replica exchange above. Replica mixing plots were created using OpenMMTools 0.21.5 (https://github.com/choderalab/openmmtools) to extract the mixing statistics from the AREX and AREST trajectories. Replica mixing was sufficient for all mutations, indicating decent phase space overlap (Supplementary Figure 10). For the REST-specific parameters, T_{max} and REST radius, all pairwise combinations of small, medium, and large values were explored for A42T and R87A. 400 K, 600 K, and 1200 K were selected for T_{max} and 0.3 nm, 0.5 nm, and 0.7 nm were selected for radius, yielding nine combinations of REST parameters. 0.5 nm and 600 K were selected for T_{max} and radius, respectively, for running complex phase AREST simulations for all mutations. The protocol used to scale the effective temperature is shown in Supplementary Figure 1. Full details and script for the AREST simulations can be found at https://github.com/choderalab/perses-barnase-barstar-paper/blob/main/scripts/04_run_repex/run_repex.py.

A.8 Free energy difference analysis

Free energy differences (ΔGs) for each phase were estimated using the MBAR implementation in pymbar 3.1.1 [60]. The MBAR estimates were initialized with zeroes for all experiments except R2Q (ACE-X-NME

bioRxiv preprint doi: https://doi.org/10.1101/2023.03.07.530278; this version posted March 8, 2023. The copyright holder for this preprint (which was not certified by peer review) is the author/funder, who has granted bioRxiv a license to display the preprint in perpetuity. It is made available under aCC-BY 4.0 International license.

phase) and two of the REST combination experiments (R87A with radius 0.5 nm and T_{max} 600 K and R87A with radius 0.7 nm and T_{max} 1200 K), which were initialized with a BAR estimate to improve solver convergence. The MBAR equations were solved using an adaptive algorithm with a solver tolerance of 1e-12. The algorithm runs both self-consistent and Newton-Raphson methods at each iteration and the method with the smallest gradient is chosen to improve numerical stability. Error bars were computed by bootstrapping the decorrelated reduced potential matrices (number of bootstraps = 200) and evaluating the free energy differences for each bootstrapped matrix with a solver tolerance of 1e-6. To assemble the decorrelated samples to feed into MBAR, the number of equilibration iterations to discard and the subsample rate were determined by applying a simple equilibration detection method [73] (implemented in OpenMMTools 0.21.5 (https://github.com/choderalab/openmmtools)) to a timeseries of the sum of the reduced potentials over all replicas. Default settings were used unless otherwise noted. For full details on ΔG estimation, see: https://github.com/choderalab/perses-barnase-barstar-paper/blob/main/scripts/05_analyze/analyze_dg.py.

The ΔG time series were generated by estimating the ΔG (using MBAR, as described above) in 1 ns intervals. The MBAR estimates were initialized with zeroes for all experiments except R2Q (ALA-X-ALA phase), which was initialized with a BAR estimate to improve solver convergence. The first 10% of samples were discarded due to equilibration and samples were selected every 5 iterations. Error bars were computed as described above. The slope (and standard error) of the last 5 ns was computed using SciPy 1.9.0's `linregress` function [108]. For the restraint experiments, residual ΔG time series plots were generated in the same manner as described above. The residual ΔG was computed as $\Delta G(t) - \Delta G(t = 10\text{ns})$, which was necessary to compare the rate of decay of the ΔGs from the non-restrained and restrained simulations, otherwise the two time series differ by an offset. For the REST parameter comparison experiments, the "true" ΔG was computed by averaging the ΔG over three replicates of 100 ns/replica AREX simulations. For comparison of AREX versus AREST, the ΔΔG discrepancy, RMSE, and MUE time series plots were computed in the same manner as described above, where the ΔΔG discrepancy for each time point was computed as $\Delta G_{\text{complex}}$ - ΔG_{apo} - $\Delta\Delta G_{\text{experiment}}$ and RMSE and MUE for each time point were computed using the $\Delta\Delta G_{\text{predicted}}$s for all 28 mutations.

ΔΔG comparison plots (forward vs negative reverse and calculated vs experiment) were generated using Cinnabar 0.3.0 (https://github.com/OpenFreeEnergy/cinnabar). For more details on generating these plots, see: https://github.com/choderalab/perses-barnase-barstar-paper/blob/main/scripts/05_analyze/0_cinnabar_plots_50ns.ipynb.

A.9 ϕ and ψ angle analysis

ϕ and ψ angle analysis was performed for 5 ns/replica A2T and R2A ACE-X-NME phase AREX simulations (Supplementary Figure 4). The ϕ and ψ angles were computed for the old positions of each replica trajectory snapshot (saved every 100 ps) for all replicas using MDTraj 1.9.7 [94]. The sine transformation was applied to the angle values in each time series. The statistical inefficiency across all replicas was computed using pymbar 3.1.1 [74]'s `statisticalInefficiencyMultiple`.

A.10 Y29 residue pair distance analysis

For the Y29 residue pair distance analysis (Supplementary Figure 7), Y29-H102 distances were computed between the closest sidechain heavy atoms and Y29-R83 and Y29-N84 distances were computed between the carbonyl oxygen of R83 or N84 and the sidechain hydroxyl oxygen of Y29. The three residue pair distances were computed for each snapshot (saved every 100 ps) of two different trajectories: 1) the old positions of Y29A AREX (50 ns/replica) at the lambda = 0 endstate and 2) the new positions of A29Y AREX (50 ns/replica) at the lambda = 1 endstate. Distances were computed using MDTraj 1.9.7 [94].

A.11 $\partial U/\partial\lambda$ correlation analysis

For the $\partial U/\partial\lambda$ correlation analysis (Figure 4B-C, E-F, Figure 5), we monitor the derivative of the potential energy with respect to the alchemical coordinate λ, $\partial U/\partial\lambda$, over time. $\partial U/\partial\lambda$ is sensitive to potential energy changes in the alchemical region but insensitive to changes in non-alchemical interactions. An ideal $\partial U/\partial\lambda$ trajectory thoroughly samples a stationary distribution (i.e., it samples all thermally accessible metastable

bioRxiv preprint doi: https://doi.org/10.1101/2023.03.07.530278; this version posted March 8, 2023. The copyright holder for this preprint (which was not certified by peer review) is the author/funder, who has granted bioRxiv a license to display the preprint in perpetuity. It is made available under aCC-BY 4.0 International license.

states multiple times), generating a sufficient number of decorrelated samples, which are required in order to produce reliable estimates of free energy differences. On the other hand, if a $\partial U/\partial\lambda$ trajectory gets stuck in one metastable state and fails to visit all metastable states multiple times, there are likely slow degrees of freedom with long correlation times that make it difficult to obtain decorrelated samples. We can quantify the degree of correlation within a time series by computing its statistical inefficiency, g.

To generate the time series for $\partial U/\partial\lambda$ and each degree of freedom, interface residues were defined as all residues within 4 Å of the other chain, with the addition of barstar residue E80 because it is one of the mutating residues in the Schreiber et al $\Delta\Delta G_{binding}$ dataset. Protein and water degrees of freedom were computed for both the old and new positions for each trajectory snapshot (saved every 100 ps) of all replica trajectories using MDTraj 1.9.7 [94]. For dihedral angles, the backbone and sidechain dihedral angles (ϕ, ψ, χ_1, χ_2, χ_3, χ_4) were computed for each interface residue. Sine and cosine transformations were applied to each angle time series and the transformation yielding the maximum magnitude in correlation to $\partial U/\partial\lambda$ was selected. For residue contacts, distances were computed between closest heavy atoms for all pairs of interface residues. For neighboring waters, water oxygens within 5 Å of any heavy atom in the mutating residue were counted. $\partial U/\partial\lambda$ was computed (for each trajectory snapshot of all replica trajectories) using numerical differentiation. Finite difference approximation with a symmetric difference quotient was used for intermediate alchemical states and Newton's difference quotient was used for alchemical endstates, with a step size of 1e-3 for both types of states. For a given mutation, to obtain the Pearson correlation coefficient (PCC) of each degree of freedom with respect to $\partial U/\partial\lambda$ across all replicas, the $\partial U/\partial\lambda$ and degree of freedom time series were separately concatenated across all replicas before computing the PCC. PCCs were computed using SciPy 1.9.0's `pearsonr` function [108] and the 95% confidence intervals were computed by bootstrapping (number of bootstraps = 200). Each bootstrapped sample was obtained by subsampling the replica indices (with replacement) and then concatenating the time series based on the subsampled replica indices. The statistical inefficiency was computed using pymbar 3.1.1 [74]'s `statisticalInefficiency` and `statisticalInefficiencyMultiple` for individual replicas and across all replicas, respectively.

A.12 Amazon Web Services (AWS) cost calculation

The GPU time was estimated using 36 replicas and the AWS costs were estimated based on the on-demand price of an Amazon EC2 P4d instance ($32.77 per hour), which has 8 NVIDIA A100 GPUs.

B Investigation of the discrepant $\Delta\Delta G_{binding}$ prediction for A29Y

With a 50 ns/replica AREX simulation, A29Y not only has a large discrepancy in $\Delta\Delta G_{binding}$ with respect to experiment (-2.11 kcal/mol), but also with respect to Y29A (1.45 kcal/mol) (Supplemntary Figure 6B and Supplementary Table 1). We hypothesize that A29Y has poor accuracy and internal consistency because the mutant tyrosine residue does not sample its most energetically favorable orientation in the barnase:barstar interface. The mutant tyrosine residue in A29Y potentially faces more difficult sampling challenges than the wild-type tyrosine residue in Y29A because the former has to be computationally modeled onto the A29 structure, whereas the positions of the latter are taken from the crystal structure and therefore the wild-type tyrosine residue is guaranteed to be in a low energy conformation. To test our hypothesis, we monitored the distances between Y29 and three nearby residues: H102, whose sidechain stacks with Y29's aromatic sidechain to form a hydrophobic interaction, R83, and N84, whose backbone carbonyl oxygens form hydrogen bonds with Y29's sidechain hydroxyl oxygen [69] (Supplementary Figure 7C). We generated time series for each of the three residue pair distances at the mutant endstate (λ = 1, where Y29 is fully interacting with its environment) of the A29Y AREX simulation and the wild-type endstate (λ = 0, where Y29 is fully interacting with its environment) of the Y29A AREX simulation. We compared the distances in each time series with the crystal structure distance and found that the Y29A wild-type endstate samples the crystal structure distance for all three residue pairs (Supplementary Figure 7A), but the A29Y mutant endstate rarely samples the crystal structure distance for two of the three residue pairs (Supplementary Figure 7B-C). These findings demonstrate that even with 50 ns of simulation time, the mutant tyrosine residue does not sample the relevant orientations that would enable it to contribute favorably to the barnase:barstar interface, which explains why the predicted $\Delta\Delta G_{binding}$ of A29Y has poor internal consistency and accuracy. We expect that

bioRxiv preprint doi: https://doi.org/10.1101/2023.03.07.530278; this version posted March 8, 2023. The copyright holder for this preprint (which was not certified by peer review) is the author/funder, who has granted bioRxiv a license to display the preprint in perpetuity. It is made available under aCC-BY 4.0 International license.

with sufficient simulation time (potentially much longer than 50 ns), the mutant tyrosine will sample the relevant orientations, eliminating the discrepancy in $\Delta\Delta G_{\text{binding}}$. Future work could involve improving the approach we use for computationally building in mutant residues.

C Investigation of the discrepant $\Delta\Delta G_{\text{binding}}$ predictions for D35A and K27A

We investigated whether the significantly discrepant D35A and K27A predictions (with 50 ns/replica AREX) are a result of failing to account for all relevant protonation states. Since arginine and glutamine do not have alternate protonation states that are easily accessible under physiological conditions, we only examined protonation state effects for D35A and K27A. We first explored the possibility that D35 may exist in both its deprotonated (ASP) and protonated (ASH) forms. We modeled D35 as ASH and ran AREX on ASH → ALA transformations in the complex (10 ns/replica), apo (10 ns/replica), and terminally-blocked (5 ns/replica) phases. We recomputed the D35A $\Delta\Delta G_{\text{binding}}$, accounting for possible interconversion between the deprotonated and protonated states (see Section C.1), and found that the $\Delta\Delta G_{\text{binding}}$ (1.65 kcal/mol) is within error of the original, deprotonated $\Delta\Delta G_{\text{binding}}$ (1.66, 95% CI: [0.57, 2.75] kcal/mol). The similar $\Delta\Delta G_{\text{binding}}$s obtained with and without accounting for multiple protonation states indicates that our original $\Delta\Delta G_{\text{binding}}$ for D35A is not discrepant because of failing to incorporate all relevant protonation states. Moreover, we observed analogous results for K27A, where the $\Delta\Delta G_{\text{binding}}$ (accounting for multiple protonation states, 3.31 kcal/mol) is within error of the original, protonated $\Delta\Delta G_{\text{binding}}$ (3.32, 95% CI: [1.80, 4.84] kcal/mol), showing that protonation state effects are not causing the discrepancy in predicted $\Delta\Delta G_{\text{binding}}$ of K27A.

C.1 Computation of $\Delta\Delta G_{\text{binding}}$s accounting for multiple protonation states

We are interested in computing the relative binding free energy, $\Delta\Delta G^{\text{binding}}_{A\to B}$, where A is the WT amino acid and B is the mutant amino acid, accounting for all relevant protonation states for both amino acids. We use D35A as an example, where A is aspartic acid (ASP) and B is alanine (ALA). ASP may exist in a deprotonated state (A) or a protonated state (AH), whereas ALA only has one state. To compute $\Delta\Delta G^{\text{binding}}_{A\to B}$, we use the thermodynamic cycles in Supplementary Figure 12.

The relative binding free energy can be defined as the difference in binding free energies between B and A:

$$\Delta\Delta G^{\text{binding}}_{A\to B} = \Delta G^{\text{binding}}_{B} - \Delta G^{\text{binding}}_{A} \tag{9}$$

The binding free energy of chemical species s (e.g., A or B), accounting for multiple protonation states, can be computed as:

$$\Delta G^{\text{binding}}_{s} = -k_B T \ln \sum_{i\in s} e^{-(\Delta G^{\text{state}}_i + \Delta G^{\text{binding}}_i)/k_B T} \tag{10}$$

where k_B is the Boltzmann constant, T is temperature, i represents a protonation state of chemical species s, ΔG^{state}_i is the protonation state free energy for state i, and $\Delta G^{binding}_i$ is the binding free energy at protonation state i. Given that the protonation state free energy can be computed as:

$$\Delta G^{\text{state}}_i(\text{pH}) = -k_B T \ln P^{state}_i(\text{pH}) \tag{11}$$

where P^{state}_i is the probability of chemical species s adopting protonation state i and pH is the pH of interest (note: we suppress the pH argument throughout the rest of the derivation), and the free energy of deprotonation can be computed as:

$$\Delta G_{AH\to A^-} = -k_B T \ln \frac{P^{state}_{A^-}}{P^{state}_{AH}}; P^{\text{state}}_{AH} + P^{state}_{A^-} = 1 \tag{12}$$

we compute the protonation state free energies as:

$$\Delta G^{state}_{A^-} = -k_B T \ln \frac{e^{-\Delta G_{AH\to A^-}/k_B T}}{1 + e^{-\Delta G_{AH\to A^-}/k_B T}} \tag{13}$$

$$\Delta G^{state}_{AH} = -k_B T \ln \frac{1}{1 + e^{-\Delta G_{AH\to A^-}/k_B T}} \tag{14}$$

bioRxiv preprint doi: https://doi.org/10.1101/2023.03.07.530278; this version posted March 8, 2023. The copyright holder for this preprint (which was not certified by peer review) is the author/funder, who has granted bioRxiv a license to display the preprint in perpetuity. It is made available under aCC-BY 4.0 International license.

If we set G_{B+X} and G_{BX} to 0, we can compute the absolute binding free energies of the deprotonated and protonated states of A as relative free energies (see Supplementary Figure 12):

$$\Delta G^{binding}_{A^-} = \Delta G_{BX\rightarrow A^-X} - \Delta G_{B+X\rightarrow A^-+X} = -\Delta G_4 - (-\Delta G_2) \quad (15)$$

$$\Delta G^{binding}_{AH} = \Delta G_{BX\rightarrow AHX} - \Delta G_{B+X\rightarrow AH+X} = -\Delta G_3 - (-\Delta G_1) \quad (16)$$

where X is the binding partner. We can compute $\Delta G^{binding}_{A^-}$ and $\Delta G^{binding}_{AH}$ from ΔG_1, ΔG_2, ΔG_3, and ΔG_4 (Supplementary Table 2). We can also compute $\Delta G^{state}_{A^-}$ and ΔG^{state}_{AH} from $\Delta G_{AH\rightarrow A^-}$ (i.e., "corrected" $\Delta G^{\text{apo}}_{AH\rightarrow A^-}$ in Supplementary Table 2). We can then feed $\Delta G^{binding}_{A^-}$, $\Delta G^{binding}_{AH}$, $\Delta G^{state}_{A^-}$, ΔG^{state}_{AH} into equation 10 to compute the binding free energy of A (i.e., ASP), accounting for both protonation states ($\Delta G^{binding}_{A}$). Finally, we can feed $\Delta G^{binding}_{A}$ into equation 9 to compute $\Delta\Delta G^{binding}_{A\rightarrow B}$. Note that since we set G_{B+X} and G_{BX} to 0, $\Delta G^{binding}_{B}$ is 0. The above calculation can be repeated for K27A where A is LYS and B is ALA.

D REST parameter selection experiments reveal that improvements in convergence are comparable across a broad range of REST parameter combinations

To run REST, the user must select the maximum effective temperature (T_{max}), which corresponds to the highest effective temperature to which the "REST" region will be scaled. The user must also choose the REST region, which we define as the mutating residue and all residues within a user-specified radius of it. The higher the T_{max} and the larger the radius, the more significantly the energy barriers will decrease and the more enhanced sampling will be. However, as one increases the T_{max} and the radius, the thermodynamic length also increases, which increases the variance of the free energy difference (ΔG) estimates. Therefore, a key challenge when applying REST is to find the optimal combination of T_{max} and radius that will decrease the correlation time of the slowest degrees of freedom while minimizing the variance of the ΔG estimate.

To explore combinations of T_{max} and radius, we chose small, medium, and large values for each of the parameters. We selected 400 K, 600 K, and 1200 K for T_{max} and 0.3 nm, 0.5 nm, and 0.7 nm for the radius. For each combination of parameters (9 total), we ran AREST for the complex phase of two representative mutations, A42T and R87A, and computed the discrepancy of the AREST $\Delta G_{\text{complex}}$ (at t = 10 ns) with respect to the "true" $\Delta G_{\text{complex}}$, which was computed from 100 ns/replica AREX. We used discrepancy as a metric to assess the efficiency of each REST parameter combination in achieving convergence to the true $\Delta G_{\text{complex}}$.

We compared the discrepancies across the REST parameter combination experiments and also against the reference (T_{max} = 300 K and no REST region) experiment. For both A42T and R87A, at the 10 ns time point, most of the REST combination $\Delta G_{\text{complex}}$s were less discrepant than the reference $\Delta G_{\text{complex}}$, indicating that AREST improves convergence more efficiently than vanilla AREX for these mutations (Supplementary Figure 8B-C). When comparing the discrepancies in $\Delta G_{\text{complex}}$s across REST combination experiments, we found that for both A42T and R87A, the discrepancies are within error of each other. The least discrepant parameter combination for both A42T and R87A is T_{max} = 600 K and radius = 0.5 nm, though this combination decreases the discrepancy only slightly better than the other combinations.

We also compared the improvements of AREST over AREX between A42T and R87A. The difference in $\Delta G_{\text{complex}}$s (at t = 10 ns) for the best REST parameter combination (T_{max} = 600 K and radius = 0.5 nm) and the reference AREX simulation is less significant for A42T (~0.5 kcal/mol) than it is for R87A (~4.5 kcal/mol). Although these results initially suggest that for A42T, AREST does not significantly improve convergence compared to AREX, if we examine the difference in discrepancies at an earlier time point (2 ns instead of 10 ns), we find that the difference is greater (~2.5 kcal/mol) than that at 10 ns (Supplementary Figure 8A-B). Therefore, AREST does improve the efficiency of $\Delta G_{\text{complex}}$ convergence for A42T, but most of the efficiency improvement occurs in the first few nanoseconds of the trajectory and afterwards, the advantages of AREST over AREX for A42T become significantly less prominent. On the other hand, for R87A, the efficiency improvement is present through at least 10 ns, perhaps even longer (Supplementary Figure 8C). Taken together, these results suggest that the same REST parameter combination can affect different mutations in the same system to varying degrees.

bioRxiv preprint doi: https://doi.org/10.1101/2023.03.07.530278; this version posted March 8, 2023. The copyright holder for this preprint (which was not certified by peer review) is the author/funder, who has granted bioRxiv a license to display the preprint in perpetuity. It is made available under aCC-BY 4.0 International license.

E Supplementary Figures and Tables

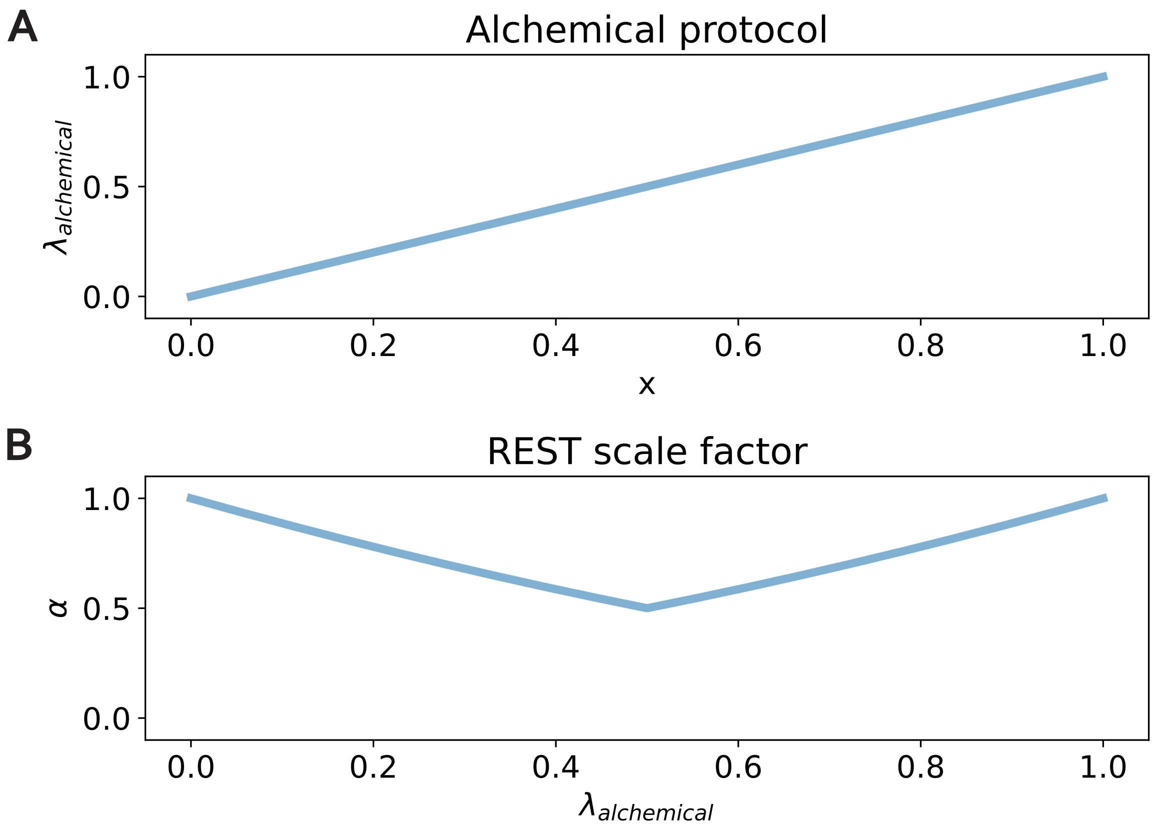

Supplementary Figure 1. Functions for defining the alchemical protocol and REST scale factor. (A) The function, $\lambda_{\text{alchemical}}(x) = x$, used for defining the alchemical protocol. **(B)** The function used for defining the REST scale factor, $\alpha(\lambda_{\text{alchemical}})$ which is proportional to $\lambda_{\text{alchemical}}\,(T_0/T_{\text{max}})$. We gradually increase the temperature from T_0 to T_{max} and back down to T_0 over the alchemical protocol, reaching T_{max} halfway through the protocol. We chose T_0 to be 300 K and T_{max} to be 600 K.

bioRxiv preprint doi: https://doi.org/10.1101/2023.03.07.530278; this version posted March 8, 2023. The copyright holder for this preprint (which was not certified by peer review) is the author/funder, who has granted bioRxiv a license to display the preprint in perpetuity. It is made available under aCC-BY 4.0 International license.

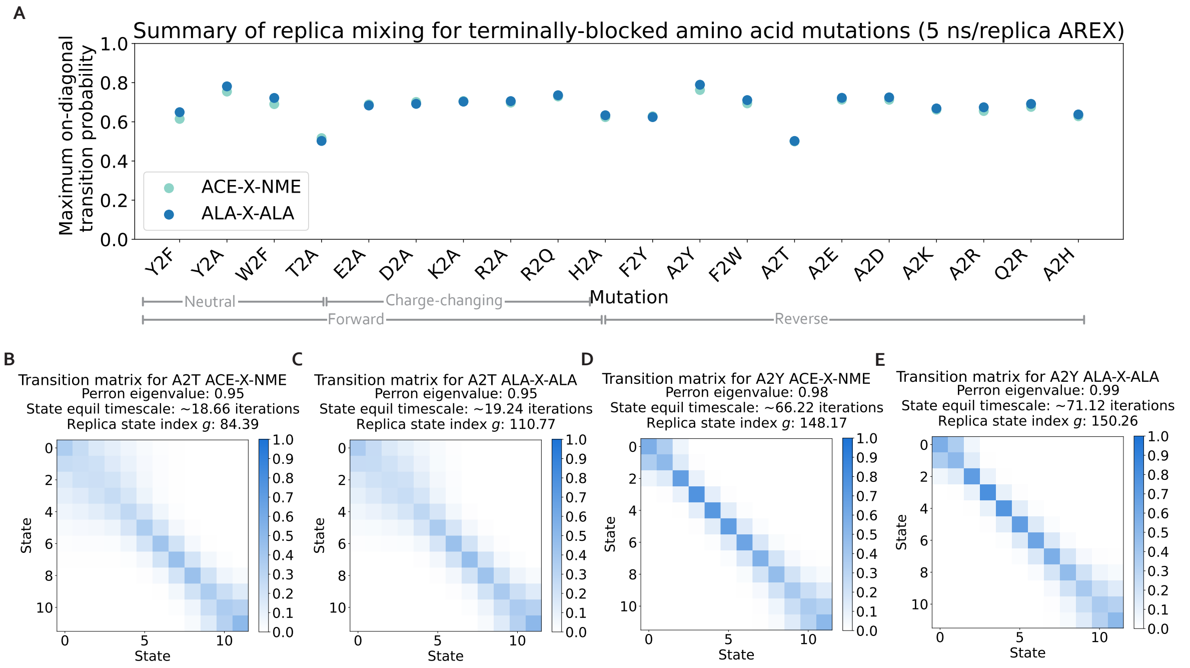

Supplementary Figure 2. Replicas mix well for all terminally-blocked amino acid alchemical replica exchange (AREX) simulations. (A) Maximum on-diagonal transition probability for the transition probability matrices of each of the 20 forward and reverse terminally-blocked amino acid mutations. Transition probability matrices generated from AREX simulations (number of states = 12 and 24 for neutral and charge mutations, respectively and simulation time = 5 ns/replica). The on-diagonal transition probability quantifies the extent to which replicas are exchanging with themselves; values close to 1 indicate there is a mixing bottleneck. Light teal indicates the ACE-X-NME phase and dark blue indicates the ALA-X-ALA phase. **(B)** The transition probability matrix for the 5 ns/replica ACE-X-NME phase AREX simulation of A2T, the mutation with the minimum value in panel A. "Perron eigenvalue" corresponds to the subdominant (second) eigenvalue and measures how well the replicas have mixed, where unity indicates poor mixing due to insufficient phase space overlap between some alchemical states. "State equil timescale" corresponds to the state equilibration timescale, which is proportional to the perron eigenvalue and estimates the number of iterations elapsed before the collection of replicas fully mix once. "Replica state index g" corresponds to the replica state index statistical inefficiency and describes how thoroughly the replicas visit all the states, where a value of unity indicates very thorough visitation of states and large values indicate poor visitation. **(C)** The transition probability matrix for the ALA-X-ALA phase AREX simulation of A2T, the mutation with the minimum value in panel A. **(D)** The transition probability matrix for the ACE-X-NME phase AREX simulation of A2Y, the mutation with the maximum value in panel A. **(E)** The transition probability for the ALA-X-ALA phase AREX simulation of A2Y, the mutation with the maximum value in panel A.

bioRxiv preprint doi: https://doi.org/10.1101/2023.03.07.530278; this version posted March 8, 2023. The copyright holder for this preprint (which was not certified by peer review) is the author/funder, who has granted bioRxiv a license to display the preprint in perpetuity. It is made available under aCC-BY 4.0 International license.

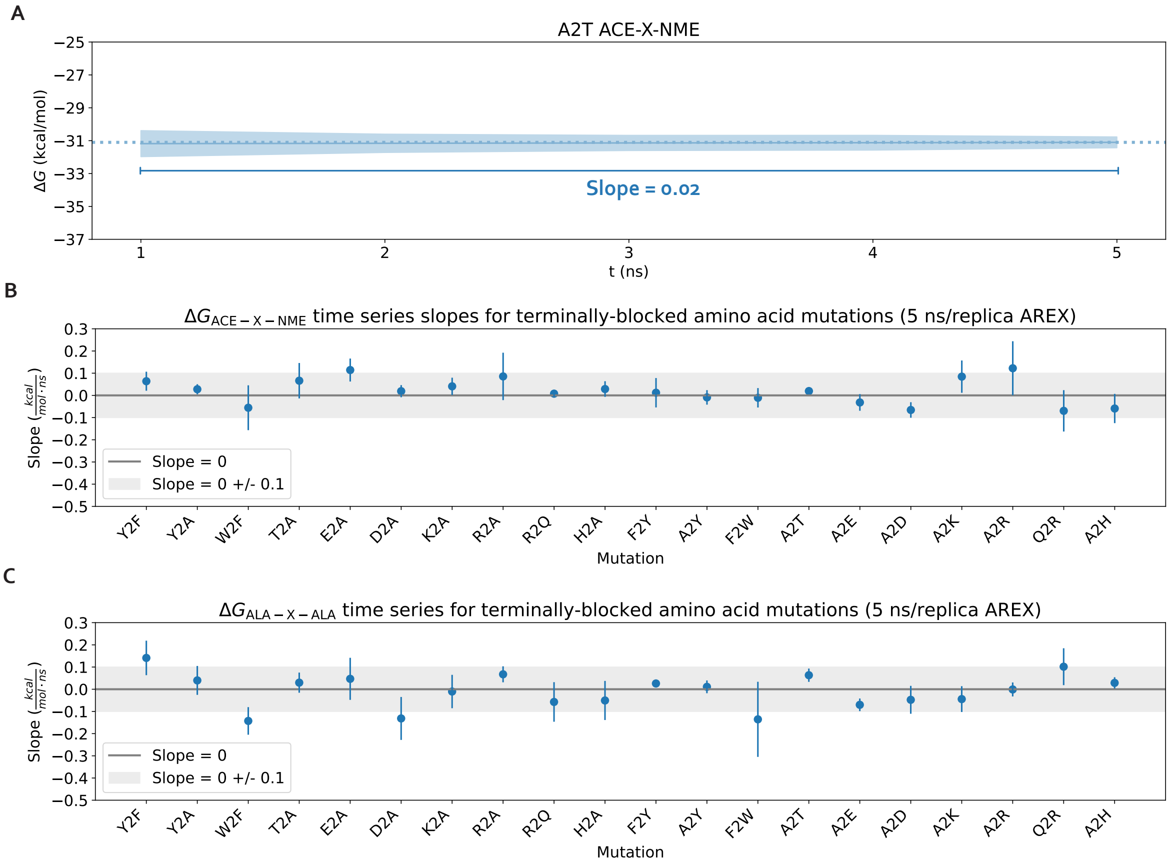

Supplementary Figure 3. The ΔG time series flatten within 5 ns for all terminally-blocked amino acid mutations. **(A)** $\Delta G_{\text{ACE-X-NME}}$ time series for A2T, shown to illustrate the data over which the slope is computed. $\Delta G_{\text{ACE-X-NME}}$ time series was generated from an AREX simulation (number of states = 12 and 24 for neutral and charge mutations, respectively and simulation time = 5 ns/replica). **(B)** Slopes of the $\Delta G_{\text{ACE-X-NME}}$ time series for each mutation are shown as blue circles. Error bars represent two standard errors and and were computed using the SciPy `linregress` function. Slopes within error of the shaded gray region (0 ± 0.1 kcal/mol/ns) are close to 0 and are therefore considered "flat." **(C)** Same as (B), but for ALA-X-ALA mutations instead of ACE-X-NME mutations.

bioRxiv preprint doi: https://doi.org/10.1101/2023.03.07.530278; this version posted March 8, 2023. The copyright holder for this preprint (which was not certified by peer review) is the author/funder, who has granted bioRxiv a license to display the preprint in perpetuity. It is made available under aCC-BY 4.0 International license.

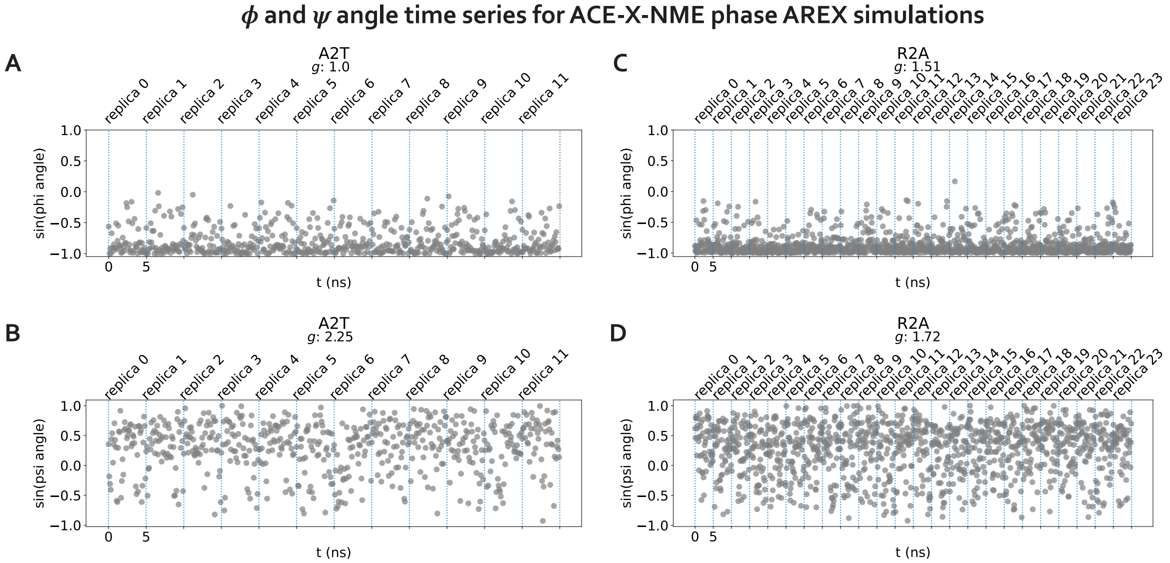

Supplementary Figure 4. The ϕ and ψ angles of two representative terminally-blocked amino acid mutations are sampled sufficiently. (A) ϕ angle time series for each replica of the A2T ACE-X-NME phase AREX simulation (number of states = 12, simulation time = 5 ns/replica). Dotted blue lines separate each replica time series. g indicates statistical inefficiency. **(B)** Same as (A), but for A2T ψ angle instead of A2T ϕ angle. **(C)** ϕ angle time series for each replica of the R2A ACE-X-NME phase AREX simulation (number of states = 24, simulation time = 5 ns/replica). **(D)** Same as (C), but for the R2A ψ angle instead of the R2A ϕ angle.

bioRxiv preprint doi: https://doi.org/10.1101/2023.03.07.530278; this version posted March 8, 2023. The copyright holder for this preprint (which was not certified by peer review) is the author/funder, who has granted bioRxiv a license to display the preprint in perpetuity. It is made available under aCC-BY 4.0 International license.

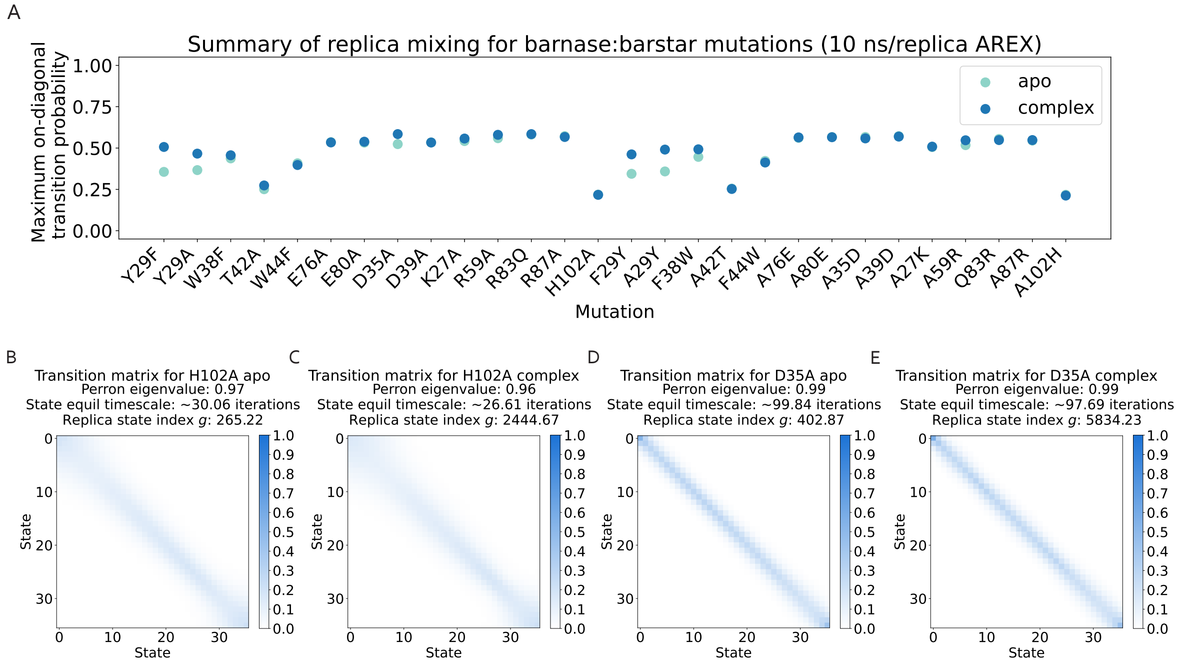

Supplementary Figure 5. Replicas mix well for all barnase:barstar alchemical replica exchange (AREX) simulations. (A) Maximum on-diagonal transition probability for the transition probability matrices of each of the 28 forward and reverse barnase:barstar mutations. Transition probability matrices generated from AREX simulations (number of states = 24 and 36 for neutral and charge mutations, respectively and simulation time = 10 ns/replica). The on-diagonal transition probability quantifies the extent to which replicas are exchanging with themselves; values close to 1 indicate there is a mixing bottleneck. Light teal indicates the apo phase and dark blue indicates the complex phase. **(B)** The transition probability matrix for the 10 ns/replica apo phase AREX simulation of H102A, the mutation with the minimum value in panel A. "Perron eigenvalue" corresponds to the subdominant (second) eigenvalue and measures how well the replicas have mixed, where unity indicates poor mixing due to insufficient phase space overlap between some alchemical states. "State equil timescale" corresponds to the state equilibration timescale, which is proportional to the perron eigenvalue and estimates the number of iterations elapsed before the collection of replicas fully mix once. "Replica state index g" corresponds to the replica state index statistical inefficiency and describes how thoroughly the replicas visit all the states, where a value of unity indicates very thorough visitation of states and large values indicate poor visitation. **(C)** The transition probability matrix for the complex phase AREX simulation of H102A, the mutation with the minimum value in panel A. **(D)** The transition probability matrix for the apo phase AREX simulation of D35A, the mutation with the maximum value in panel A. **(E)** The transition probability matrix for the complex phase AREX simulation of D35A, the mutation with the maximum value in panel A.

bioRxiv preprint doi: https://doi.org/10.1101/2023.03.07.530278; this version posted March 8, 2023. The copyright holder for this preprint (which was not certified by peer review) is the author/funder, who has granted bioRxiv a license to display the preprint in perpetuity. It is made available under aCC-BY 4.0 International license.

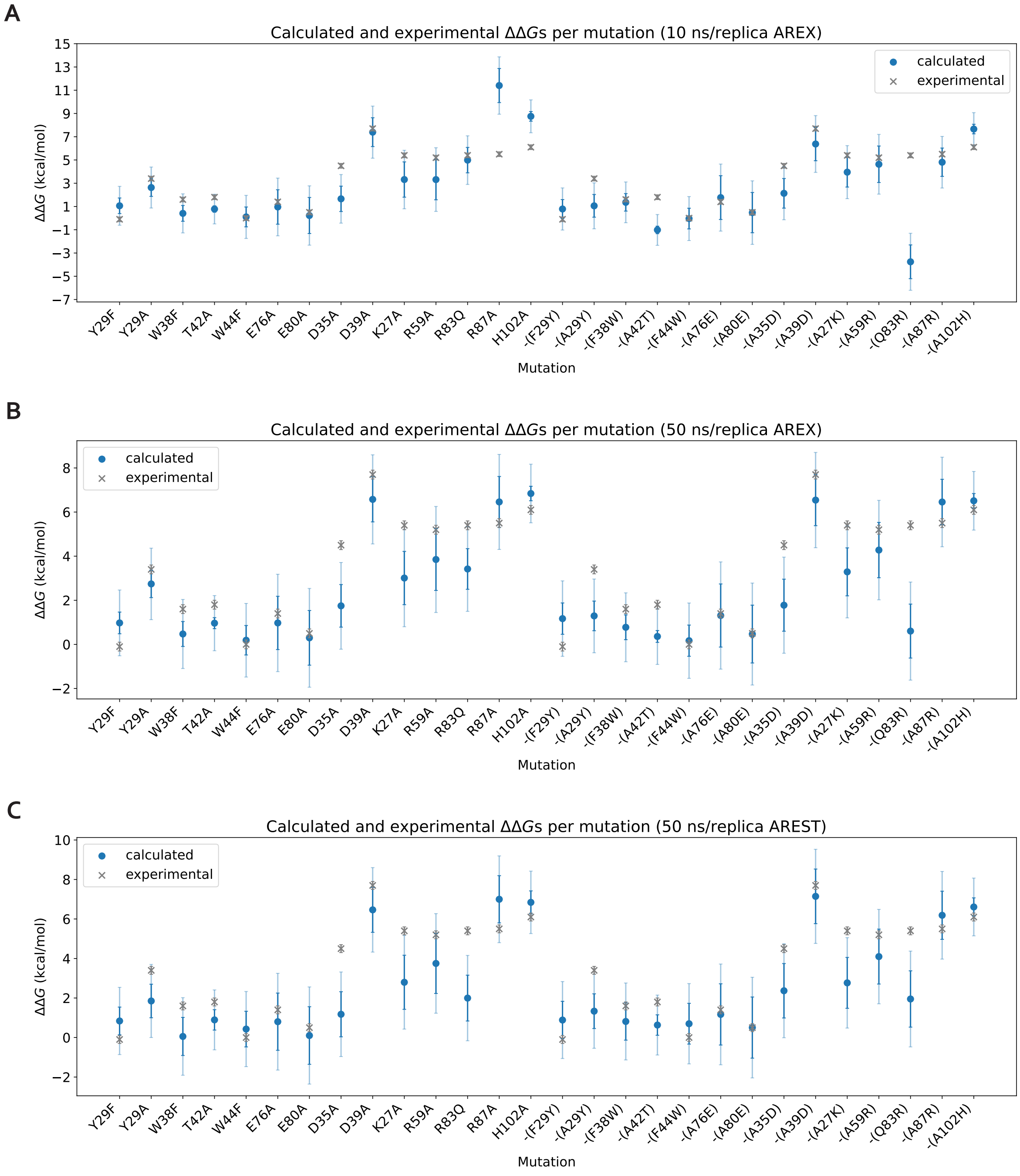

Supplementary Figure 6. Calculated ΔΔGs for barnase:barstar mutations show decent agreement with experimental ΔΔGs with 10 ns/replica AREX and agreement improves with 50 ns/replica AREX and AREST simulations. The data in this figure is the same data as in Figure 6B, D, F and is shown here in an alternate representation for clarity. **(A)** Calculated and experimental ΔΔGs per mutation for 10 ns/replica alchemical replica exchange (AREX). Dark blue error bars represent two standard errors and were computed by bootstrapping the decorrelated reduced potential matrices 200 times. Light blue error bars represent two standard errors ± 1 kcal/mol, shown to help determine whether the calculated ΔΔG is within 1 kcal/mol of experimental ΔΔG. Gray error bars indicate two standard errors and were taken from Schreiber et al. [67] **(B)** Same as (A) but for 50 ns/replica AREX. **(C)** Same as (A) but for 50 ns/replica AREST.

bioRxiv preprint doi: https://doi.org/10.1101/2023.03.07.530278; this version posted March 8, 2023. The copyright holder for this preprint (which was not certified by peer review) is the author/funder, who has granted bioRxiv a license to display the preprint in perpetuity. It is made available under aCC-BY 4.0 International license.

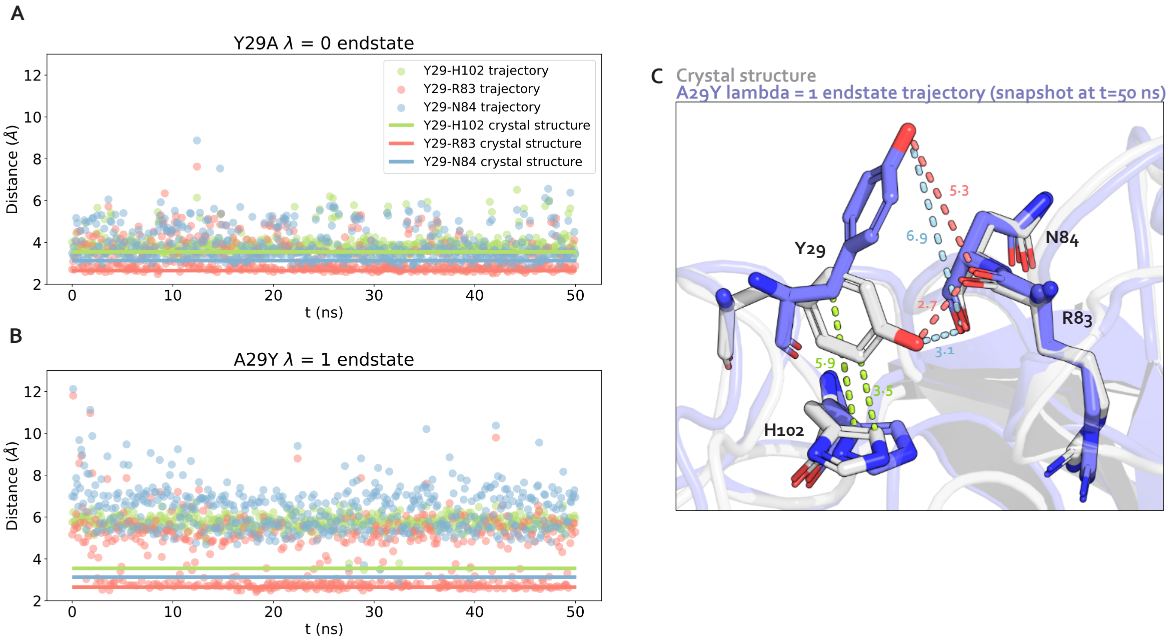

Supplementary Figure 7. During a 50 ns/replica AREX simulation, the mutant tyrosine residue of A29Y rarely finds its most energetically favorable orientation in the barnase:barstar interface. (A) Distance time series for three residue pairs: Y29-H102 (green), Y29-R83 (pink), and Y29-N84 (blue) in the Y29A $\lambda = 0$ endstate trajectory (simulation time between frames: 100 ps, total simulation time: 50 ns). Horizontal lines represent the crystal structure (PDB ID: 1BRS) distance for each residue pair. **(B)** Same as (A) but for the A29Y $\lambda = 1$ endstate trajectory instead of the Y29A $\lambda = 0$ endstate trajectory. **(C)** Structural representation of Y29-H102, Y29-R83, and Y29-N84 residue pairs for the crystal structure (light gray) and the last snapshot (t = 50 ns) of the A29Y $\lambda = 1$ endstate trajectory (purple). Distances (in Å) between Y29-H102 (green), Y29-R83 (pink), and Y29-N84 (blue) shown as dotted lines. Nitrogen atoms in dark blue and oxygen atoms in red.

bioRxiv preprint doi: https://doi.org/10.1101/2023.03.07.530278; this version posted March 8, 2023. The copyright holder for this preprint (which was not certified by peer review) is the author/funder, who has granted bioRxiv a license to display the preprint in perpetuity. It is made available under aCC-BY 4.0 International license.

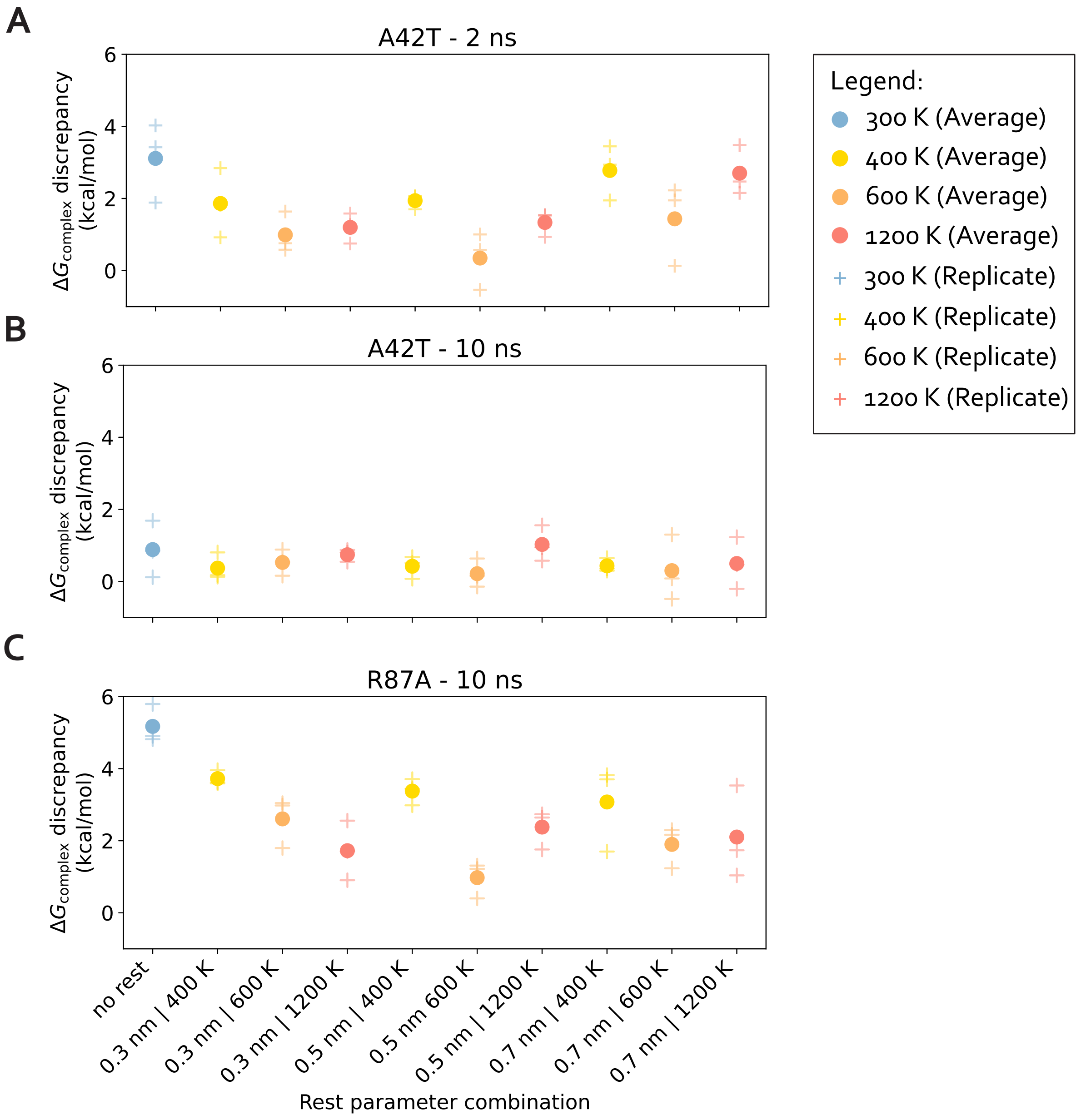

Supplementary Figure 8. The REST parameter combinations show comparable convergence, but 0.5 nm and 600 K is (marginally) the best for both A42T and R87A. (A) Comparison of different combinations of two REST parameters: maximum temperature (T_{max}) and radius. For each combination, the discrepancy of the complex phase AREST free energy difference at 2 ns with respect to the "true" free energy difference ($\Delta G^{AREST}_{t=2ns} - \Delta G^{AREST}_{t=100ns}$) was computed. Blue markers represent the case where no REST was used (i.e., T_{max} = 300 K), yellow markers represent T_{max} = 400 K, orange markers represent T_{max} = 600 K, and red markers represent T_{max} = 1200 K. Circles represent the mean discrepancy across 3 replicates and plus signs represent the discrepancy for each individual replicate. **(B)** Same as (A), but with the discrepancy computed at 10 ns instead of 2 ns. **(C)** Same as (A), but using R87A instead of A42T and with the discrepancy computed at 10 ns instead of 2 ns.

bioRxiv preprint doi: https://doi.org/10.1101/2023.03.07.530278; this version posted March 8, 2023. The copyright holder for this preprint (which was not certified by peer review) is the author/funder, who has granted bioRxiv a license to display the preprint in perpetuity. It is made available under aCC-BY 4.0 International license.

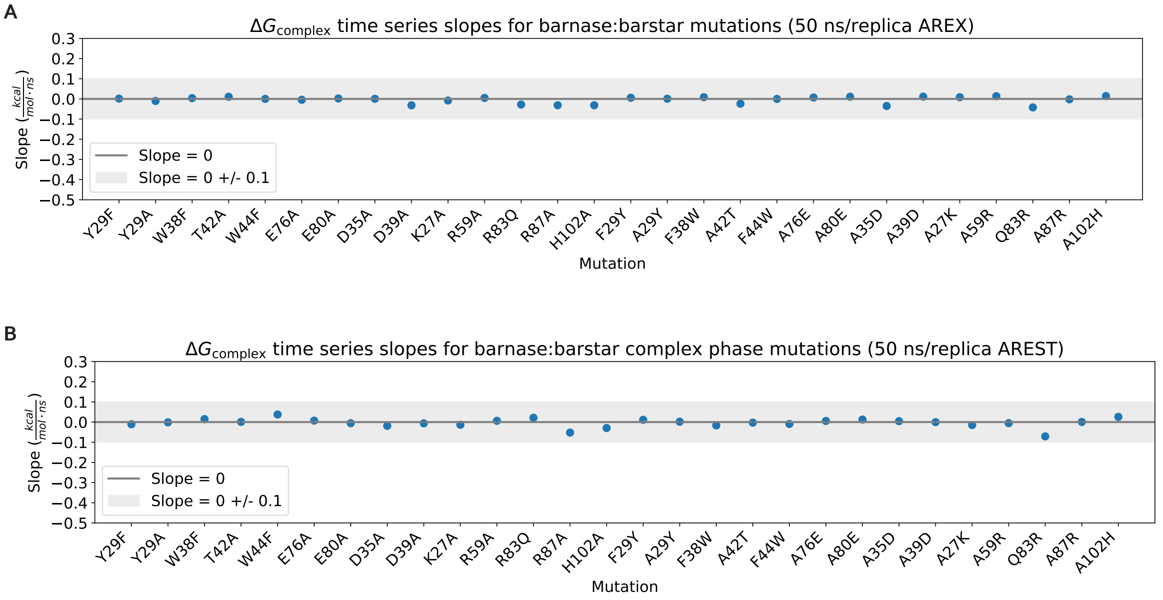

Supplementary Figure 9. The $\Delta G_{complex}$ time series converge with long alchemical replica exchange (AREX) and alchemical replica exchange with solute tempering (AREST) simulations. (A) Slopes of the last 5 ns of the $\Delta G_{complex}$ time series for each barnase:barstar mutation are shown as blue circles. $\Delta G_{complex}$ time series were generated from complex phase AREX simulations (number of states = 24 and 36 for neutral and charge mutations, respectively and simulation time = 50 ns/replica). Error bars represent 2 standard errors and were computed using the SciPy `linregress` function. Slopes within error of the shaded gray region (0 ± 0.1 kcal/mol/ns) are close to 0 and are therefore considered "flat." **(B)** Same as (A), but for AREST complex phase simulations (number of states = 24 and 36 for neutral and charge mutations, respectively and simulation time = 50 ns/replica) instead of 50 ns/replica AREX complex phase simulations.

bioRxiv preprint doi: https://doi.org/10.1101/2023.03.07.530278; this version posted March 8, 2023. The copyright holder for this preprint (which was not certified by peer review) is the author/funder, who has granted bioRxiv a license to display the preprint in perpetuity. It is made available under aCC-BY 4.0 International license.

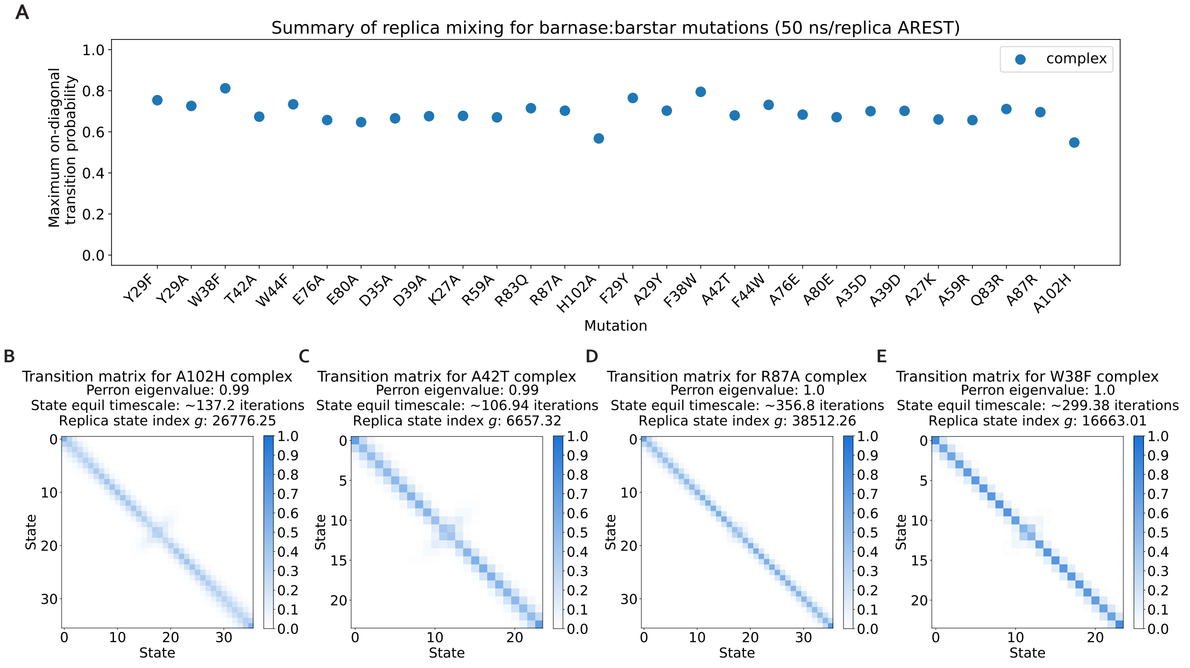

Supplementary Figure 10. Replica mixing is sufficient for all barnase:barstar alchemical replica exchange with solute tempering (AREST) simulations. (A) Maximum on-diagonal transition probability for the transition probability matrices of each of the 28 forward and reverse barnase:barstar mutations. Transition probability matrices generated from complex phase AREST simulations (number of states = 24 and 36 for neutral and charge mutations, respectively and simulation time = 50 ns/replica). The on-diagonal transition probability quantifies the extent to which replicas are exchanging with themselves; values close to 1 indicate there is a mixing bottleneck. **(B)** The transition probability matrix for the 50 ns/replica complex phase AREST simulation of A102H, the mutation with the minimum value in panel A. "Perron eigenvalue" corresponds to the subdominant (second) eigenvalue and measures how well the replicas have mixed, where unity indicates poor mixing due to insufficient phase space overlap between some alchemical states. "State equil timescale" corresponds to the state equilibration timescale, which is proportional to the perron eigenvalue and estimates the number of iterations elapsed before the collection of replicas fully mix once. "Replica state index g" corresponds to the replica state index statistical inefficiency and describes how thoroughly the replicas visit all the states, where a value of unity indicates very thorough visitation of states and large values indicate poor visitation. **(C)** The transition probability matrix for the complex phase AREST simulation of A42T, a mutation with a maximum on-diagonal transition probability close to the mean in panel A. **(D)** The transition probability matrix for the complex phase AREST simulation of R87A, a mutation with a maximum on-diagonal transition probability close to the mean in panel A. **(E)** The transition probability matrix for the complex phase AREST simulation of W38F, the mutation with the maximum value in panel A.

bioRxiv preprint doi: https://doi.org/10.1101/2023.03.07.530278; this version posted March 8, 2023. The copyright holder for this preprint (which was not certified by peer review) is the author/funder, who has granted bioRxiv a license to display the preprint in perpetuity. It is made available under aCC-BY 4.0 International license.

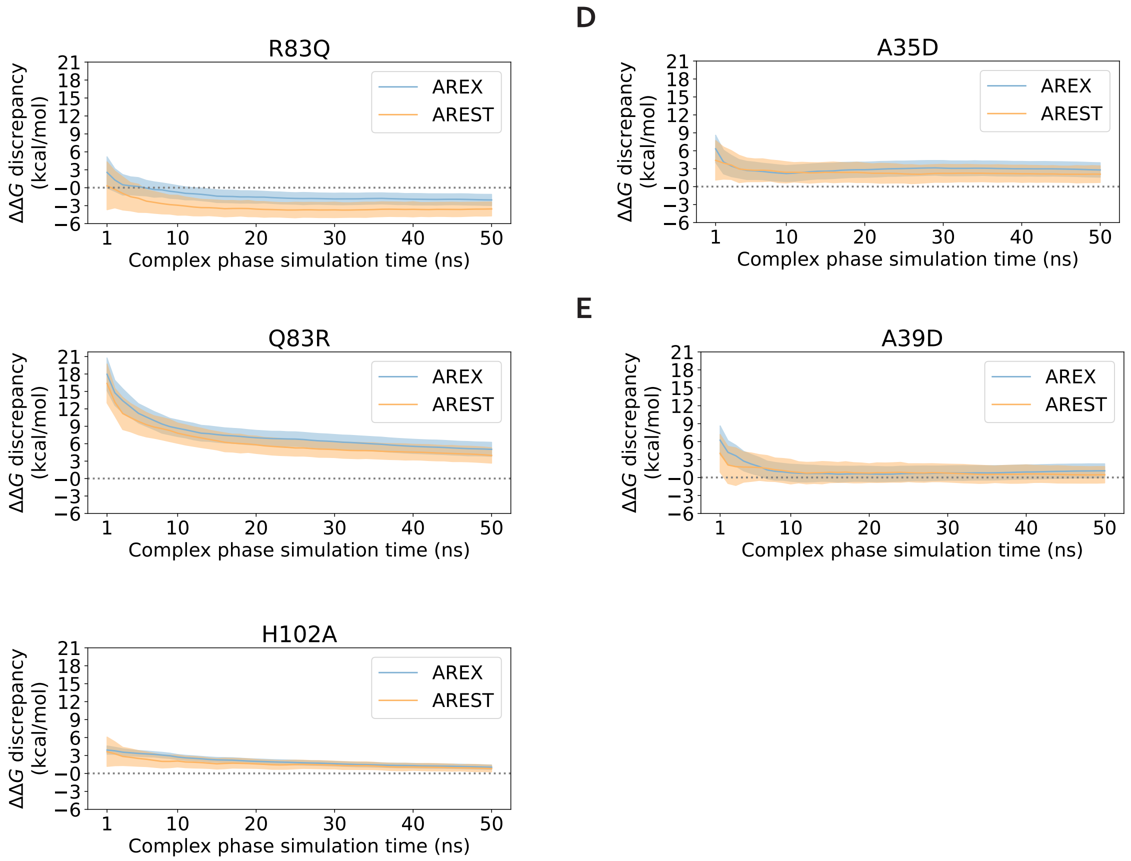

Supplementary Figure 11. With 50 ns/replica simulation time, AREST does not significantly improve convergence over AREX for most barnase:barstar mutations with slow $\Delta G_{complex}$ convergence. (A)-(E): $\Delta\Delta G$ discrepancy (with respect to experiment) time series for significantly discrepant mutations R83Q, Q83R, H102A, A35D, and A39D. The discrepancy was computed as $\Delta G_{complex} - \Delta G_{apo} - \Delta\Delta G_{experiment}$, where $\Delta G_{complex}$ corresponds to the (AREX or AREST) complex phase ΔG at a particular time point, ΔG_{apo} corresponds to the apo phase ΔG computed from a 10 ns/replica AREX simulation, and $\Delta\Delta G_{experiment}$ is the experimental value from Schreiber et al [67]. Alchemical replica exchange (AREX) time series shown in blue and alchemical replica exchange with solute tempering (AREST, with radius = 0.5 nm, T_{max} = 600 K) time series shown in orange. For AREX and AREST simulations, number of states = 24 and 36 for neutral and charge-changing mutations, respectively, and simulation time = 50 ns/replica. Shaded regions represent ± two standard errors. Gray dashed line indicates $\Delta\Delta G$ discrepancy = 0.

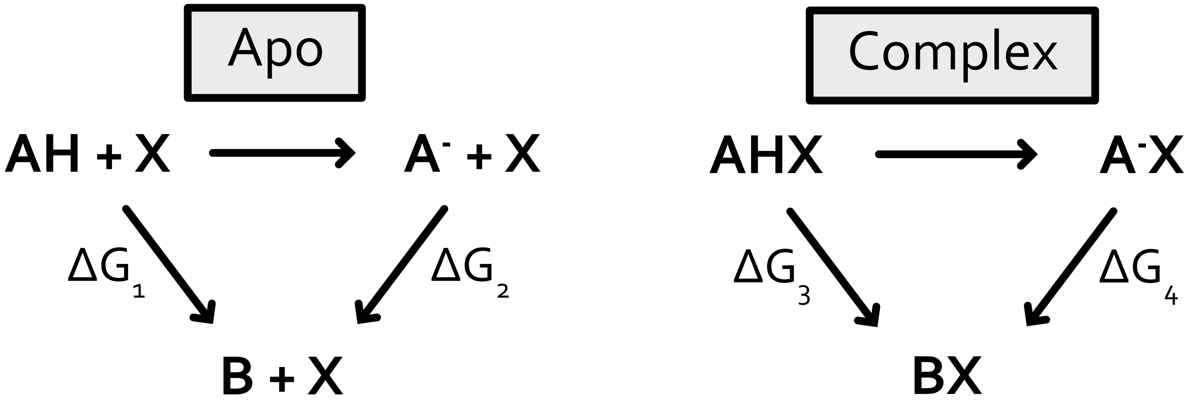

Supplementary Figure 12. Thermodynamic cycles for computing the relative binding free energy, $\Delta\Delta G^{binding}_{A \rightarrow B}$, accounting for multiple protonation states. A^- represents the deprotonated form of the WT amino acid, AH represents the protonated form of the WT amino acid, B represents the mutant amino acid, X represents the binding partner.

bioRxiv preprint doi: https://doi.org/10.1101/2023.03.07.530278; this version posted March 8, 2023. The copyright holder for this preprint (which was not certified by peer review) is the author/funder, who has granted bioRxiv a license to display the preprint in perpetuity. It is made available under aCC-BY 4.0 International license.

Mutation	Predicted ΔΔG (kcal/mol)	Error (kcal/mol)	Experimental ΔΔG (kcal/mol)	Complex phase simulation time (ns/replica)	Apo phase simulation time (ns/replica)	Mutation direction
Y29F	0.97	0.25	-0.1	50	10	forward
Y29A	2.74	0.31	3.4	50	10	forward
W38F	0.47	0.28	1.6	50	10	forward
T42A	0.96	0.12	1.8	50	10	forward
W44F	0.19	0.33	0	50	10	forward
E76A	0.97	0.6	1.4	50	10	forward
E80A	0.3	0.62	0.5	50	10	forward
D35A	1.75	0.48	4.5	50	10	forward
D39A	6.58	0.51	7.7	50	10	forward
K27A	3.01	0.6	5.4	50	10	forward
R59A	3.85	0.7	5.2	50	10	forward
R83Q	3.42	0.46	5.4	50	10	forward
R87A	6.46	0.58	5.5	50	10	forward
H102A	6.84	0.16	6.1	50	10	forward
F29Y	-1.17	0.36	0.1	50	10	reverse
A29Y	-1.29	0.34	-3.4	50	10	reverse
F38W	-0.78	0.28	-1.6	50	10	reverse
A42T	-0.36	0.13	-1.8	50	10	reverse
F44W	-0.17	0.35	0	50	10	reverse
A76E	-1.31	0.72	-1.4	50	10	reverse
A80E	-0.47	0.66	-0.5	50	10	reverse
A35D	-1.78	0.59	-4.5	50	10	reverse
A39D	-6.54	0.58	-7.7	50	10	reverse
A27K	-3.29	0.54	-5.4	50	10	reverse
A59R	-4.28	0.63	-5.2	50	10	reverse
Q83R	-0.6	0.61	-5.4	50	10	reverse
A87R	-6.46	0.51	-5.5	50	10	reverse
A102H	-6.51	0.16	-6.1	50	10	reverse
Q83R	-2.76	0.59	-5.4	100	10	reverse

Supplementary Table 1. $\Delta\Delta G_{binding}$s for barnase:barstar mutations computed from AREX simulations (with 50 ns/replica and 10 ns/replica simulation time for complex and apo phases, respectively). "Error" corresponds to one standard error and and were computed by bootstrapping the decorrelated reduced potential matrices 200 times. The last row corresponds to the $\Delta\Delta G_{binding}$ for Q83R where the complex phase simulation time was 100 ns/replica. CSV file available at https://github.com/choderalab/perses-barnase-barstar-paper/blob/main/data/table_50ns_arex.csv.

bioRxiv preprint doi: https://doi.org/10.1101/2023.03.07.530278; this version posted March 8, 2023. The copyright holder for this preprint (which was not certified by peer review) is the author/funder, who has granted bioRxiv a license to display the preprint in perpetuity. It is made available under aCC-BY 4.0 International license.

D35A

Phase	Mutation	ΔG (kT)	Error (kT)	Corresponding label in Supplementary Figure 12
Experiment	ASH->ASP	-9.44	N/A	
ACE-X-NME	ASP->ALA	5.02	1.20	
	ASH->ALA	90.83	0.23	
	ASH->ASP	85.81	1.22	
Apo	ASP->ALA	-0.84	0.82	ΔG_2
	ASH->ALA	91.03	0.16	ΔG_1
	ASH->ASP	91.87	0.84	
	ASH->ASP corrected	-3.37	1.48	
Complex	ASP->ALA	1.94	0.59	ΔG_4
	ASH->ALA	92.75	0.18	ΔG_3
	ASH->ASP	90.81	0.62	
	ASH->ASP corrected	-4.44	1.37	

K27A

Phase	Mutation	ΔG (kT)	Error (kT)	Corresponding label in Supplementary Figure 12
Experiment	LYS->LYN	5.85	N/A	
ACE-X-NME	LYN->ALA	27.74	0.42	
	LYS->ALA	-119.47	1.31	
	LYS->LYN	-147.21	1.38	
Apo	LYN->ALA	27.75	0.29	ΔG_2
	LYS->ALA	-112.52	0.96	ΔG_1
	LYS->LYN	-140.27	1.00	
	LYS->LYN corrected	12.79	1.71	
Complex	LYN->ALA	29.91	0.31	ΔG_4
	LYS->ALA	-106.95	0.93	ΔG_3
	LYS->LYN	-136.86	0.98	
	LYS->LYN corrected	16.20	1.69	

Supplementary Table 2. ΔGs for computation of $\Delta\Delta G_{binding}$s (accounting for multiple protonation states) for D35A and K27A. The "experiment" ΔGs are shown in yellow and were computed as $\Delta G = k_B T(\text{p}K_a - \text{pH}) \ln 10$ using a pH of 8.0 [67]. The ΔGs for mutations to alanine are shown in white and were computed from 5 ns/replica ACE-X-NME, 10 ns/replica apo, or 10 ns/replica complex phase simulations. The ΔGs for deprotonation ($AH \rightarrow A^{-}$) are shown in blue and were computed by subtracting pairs of ΔGs for mutations to alanine (white rows). The "corrected" $\Delta G_{AH \rightarrow A^-}$s are shown in green and were computed according to equation 1 of Mongan et al [109] (using the $\Delta G_{AH \rightarrow A^-}$s in the yellow and blue rows in the table). "Error" corresponds to one standard error and was computed by bootstrapping the decorrelated reduced potential matrices 200 times (white rows). The bootstrapped uncertainties were propagated for the blue and green rows. The XLSX file is available at https://github.com/choderalab/perses-barnase-barstar-paper/blob/main/data/D35A_K27A.xlsx.
